# Optimization of potential targets for antidepressant Chinese medicines: AI and multi-omics methods

**DOI:** 10.1186/s13020-025-01320-w

**Published:** 2026-02-11

**Authors:** Chaofang Lei, Zhigang Chen, Chongyang Ma, Le Xie, Dahua Wu, Jianbei Chen, Jiaxu Chen

**Affiliations:** 1 Department of Neurology, Hunan Provincial Hospital of Integrated Traditional Chinese and Western Medicine (Affiliated Hospital of Hunan Academy of Traditional Chinese Medicine), Changsha, China; 2 Department of Neurology, Dongfang Hospital, Beijing University of Chinese Medicine, Beijing, China; 3 School of Traditional Chinese Medicine, Capital Medical University, Beijing, China; 4 School of Chinese Medicine, Hubei University of Chinese Medicine, Wuhan, China; 5 School of Traditional Chinese Medicine, Beijing University of Chinese Medicine, Beijing, China; 6 Guangzhou Key Laboratory of Formula-Pattern of Traditional Chinese Medicine, School of Traditional Chinese Medicine, Jinan University, Guangzhou, China

**Keywords:** Artificial intelligence, Omics, Traditional Chinese medicine, Potential targets, Drug discovery, Depression

## Abstract

**Abstract:**

The complexity of traditional Chinese medicine (TCM), with its numerous components, targets, and varying efficacy, presents challenges for current evaluation methods. Most existing methods rely on single, qualitative indicators, which provide limited insight into the overall quality. These methods fail to fully capture the intrinsic quality, efficacy, and safety of Chinese medicine, highlighting the need for more advanced biological evaluation techniques. Target-based drug discovery has become the primary approach in pharmaceutical research and development, where drug targets play a crucial role in guiding the entire process. As our understanding deepens, integrating artificial intelligence (AI) with multi-omics technologies has opened new possibilities for enhancing treatment precision. AI’s efficiency in identifying drug targets marks a significant leap forward in drug discovery, facilitating the modernization of the drug development process. Meanwhile, omics technologies offer distinct advantages, such as comprehensive controllability, strong correlations with clinical efficacy and safety, and a holistic view of the overall quality of Chinese medicine. These technologies provide an effective and rational approach for evaluating the quality of Chinese medicine and are instrumental in developing quality control systems for TCM. Consequently, combining AI with multi-omics methods is poised to become a key direction for future research into the discovery of targets for antidepressant Chinese medicine.

**Graphical Abstract:**

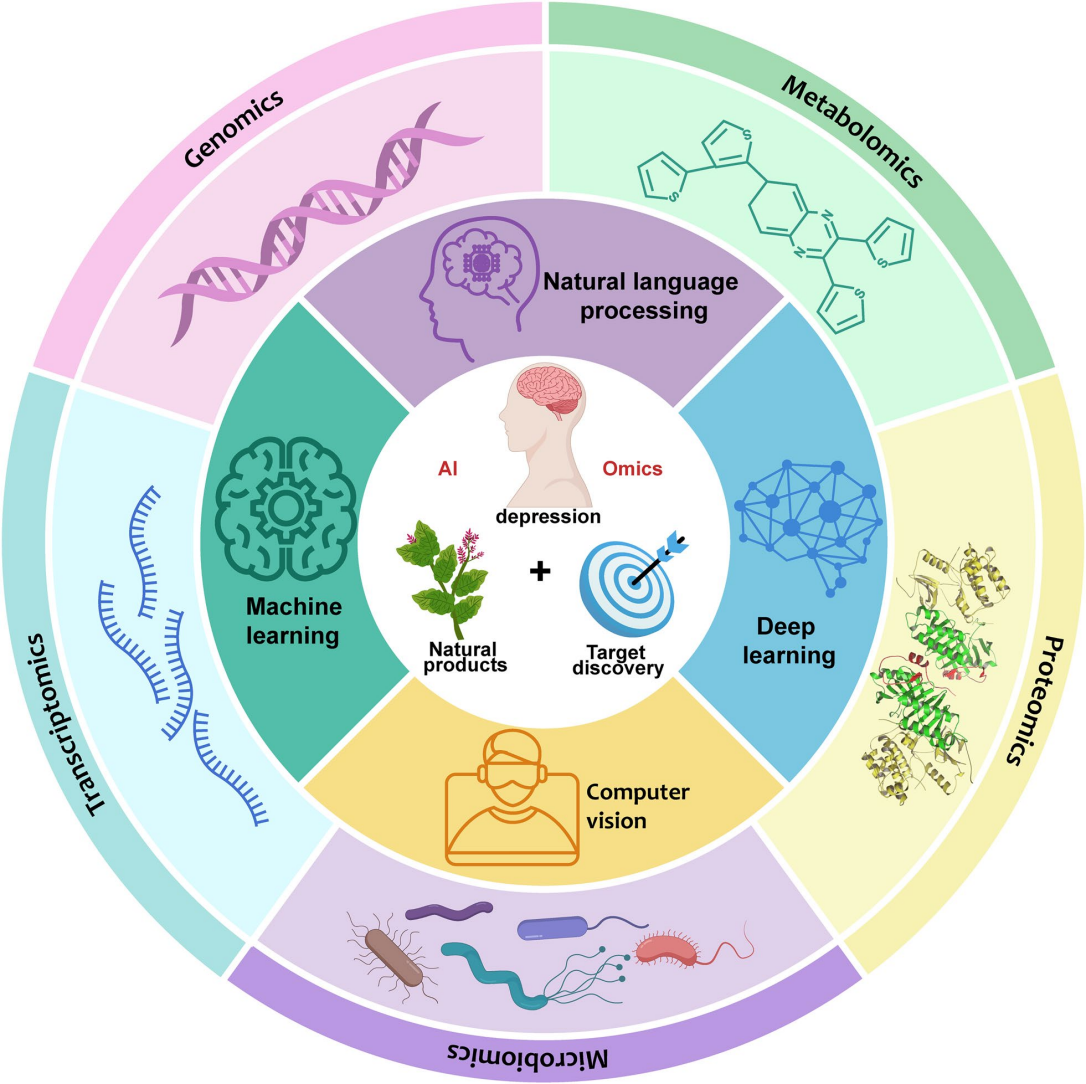

## Introduction

Depression is a global mental health disorder that significantly impairs the quality of life for millions and imposes a substantial economic burden on society. According to the World Health Organization, depression ranks among the leading causes of long-term disability, with its prevalence increasing annually. It is now the second most common cause of disability worldwide [1]. Although traditional antidepressants, such as selective serotonin reuptake inhibitors (SSRIs) and monoamine oxidase inhibitors (MAOIs), can alleviate depressive symptoms to some extent, they have limitations in efficacy and are associated with significant side effects. Many patients experience poor efficacy and adverse reactions when using these drugs [2].

In response to these challenges, increasing attention has been given to Chinese medicine as a potential alternative treatment for depression. Chinese medicine, with its multi-component and multi-target approach, offers unique advantages for managing depression. Central to Chinese medicine is the "holistic view" and "syndrome differentiation and treatment," which assert that depression is closely linked to an individual’s physical condition, emotional state, and environmental factors. As a result, Chinese medicine seeks to address depression through a multi-faceted regulatory approach [3]. For example, compound prescriptions like Chaihu Shugan Powder and Xiaoyao Powder are commonly used in clinical settings and have demonstrated both efficacy and safety in treating depression [4, 5]. These compounds typically regulate several biological pathways, including the monoamine neurotransmitter system and the hypothalamic–pituitary–adrenal (HPA) axis, thereby effectively alleviating depressive symptoms [6, 7].

AI-driven models can efficiently extract features from biological data and accurately model molecular interactions, significantly improving the precision of drug target prediction [8]. The integration of AI into drug target discovery has streamlined the traditionally labor-intensive drug development process, reducing research costs and accelerating timelines [9]. Specifically, AI applies machine learning algorithms to identify potential drug targets and elucidate mechanisms of action, offering new strategies for screening and validating antidepressant components in Chinese medicine [10]. It also helps uncover the complex interactions between Chinese medicine components and their molecular targets [11]. One of AI’s key advantages in this field is its ability to facilitate the design of multi-target drugs. Traditional drug discovery often focuses on a single target, but AI makes it feasible to identify multiple therapeutic targets simultaneously. This capability enables the development of drug combinations with synergistic effects, a strategy known as “multi-drug targeted pharmacology.” Studies have demonstrated that multi-target drugs can more effectively address complex diseases [12].

Advances in omics technologies have enabled researchers to investigate the components and mechanisms of traditional Chinese medicine (TCM) with greater depth and systematic precision. Integrating these technologies allows for a comprehensive understanding of the complex biological underpinnings of depression [13, 14]. In drug development, omics approaches play a critical role in identifying drug targets and evaluating both safety and efficacy. By analyzing how drugs affect cellular and tissue-level processes, researchers can elucidate mechanisms of action and refine strategies for drug design and screening. Moreover, omics technologies offer new avenues for drug repurposing within Chinese medicine. By applying omics analyses to existing compounds, researchers can uncover potential antidepressant effects and explore underlying mechanisms [2, 15]. This approach accelerates drug discovery and expands therapeutic options for clinical application. Spatial omics further enhance this understanding by providing essential spatial context regarding the functions and interactions of drug targets. Through high-resolution analysis of the spatial distribution of biomolecules in tissues, researchers can gain deeper insights into the pathological mechanisms of depression and their complex associations with drug responses. In conclusion, the complexity of depression and the multifaceted nature of TCM have driven researchers to seek more effective therapeutic strategies. In recent years, the integration of AI and omics technologies with TCM research has emerged as a prominent and promising direction. Although designing multi-target drugs presents significant challenges, combining AI with omics technologies has made this process more systematic, efficient, and data-driven. This integrated approach offers novel insights into the mechanisms of antidepressant compounds in Chinese medicine and facilitates the identification of relevant drug targets. It also enhances our understanding of the pathophysiology of depression and supports the development of more precise and holistic treatment strategies—reflecting the core principle of TCM's "holistic view." Continued research in this area holds the potential to improve therapeutic outcomes for depression and significantly enhance patients’ quality of life.

## Application of AI in antidepressant Chinese medicine research

### Overview of AI technology

#### Advances in machine learning

Machine learning, a core discipline within artificial intelligence, has advanced rapidly in recent years. With the exponential growth of data and improvements in computational power, its applications have expanded across diverse domains—particularly in medicine, where its impact continues to grow [16–18]. In this article, we explore the major types of machine learning and recent trends in the field, including supervised and unsupervised learning, the opportunities and limitations of reinforcement learning, and emerging developments in transfer learning.

##### Supervised learning and unsupervised learning

Supervised and unsupervised learning represent the two primary branches of machine learning [19, 20]. Supervised learning relies on labeled datasets and learns to map input variables to corresponding outputs. Common supervised algorithms include linear regression, decision trees, support vector machines, and neural networks [21]. These methods have shown strong performance in various medical applications, particularly in disease prediction and diagnosis [22]. For example, deep learning, especially in supervised settings, has demonstrated remarkable success in medical image analysis, including tasks such as classification, detection, and segmentation. Research reports indicate that deep learning models not only outperform traditional image processing techniques but also, in some aspects, have diagnostic accuracy comparable to that of human experts [23–25].

Unsupervised learning, unlike supervised learning, does not rely on labeled data. It primarily focuses on data clustering and dimensionality reduction, aiming to uncover underlying structures and patterns in the data [21]. Common unsupervised learning algorithms include K-means clustering, Principal Component Analysis (PCA), and autoencoders [21]. In recent years, unsupervised learning has proven particularly advantageous for handling large-scale datasets, with significant applications in fields such as image processing and natural language processing [26, 27]. For instance, autoencoders, through feature learning, enable researchers to extract meaningful features from unlabeled data, thereby enhancing the model's generalization capability [28].

As technological advancements continue, the distinction between supervised and unsupervised learning has become less clear. Many studies now explore hybrid approaches, such as semi-supervised and weakly supervised learning. These methods combine a small amount of labeled data with a large volume of unlabeled data, significantly improving both the efficiency and accuracy of the model [29].

##### Applications and challenges of reinforcement learning

Reinforcement learning, a key branch of machine learning, enables agents to learn optimal strategies through interactions with their environment [30, 31]. Its core mechanism involves guiding agent behavior using a system of rewards and penalties to achieve predefined goals [32]. In recent years, reinforcement learning has shown great promise in medical image analysis, omics research, and biological imaging [33, 34]. For example, a reinforcement learning model informed by human neuroimaging data and based on reward prediction error calculations has provided in vivo evidence that striatal dopaminergic signaling interacts with cortical regional networks to drive task-based learning strategies. Rather than merely reflecting reward outcomes, these findings underscore the model's potential for complex decision-making tasks [35].

Despite its potential, reinforcement learning faces several practical challenges. First, it often requires large volumes of interactive data for effective training, which can be impractical in high-risk domains such as medicine and mechanical engineering [36]. Second, training tends to be unstable and highly sensitive to environmental changes, which can cause rapid performance degradation [37]. Third, the limited interpretability of reinforcement learning models restricts their adoption in applications that demand transparency and reliability [38].

To overcome these limitations, researchers have developed several enhancements. For instance, techniques like model predictive control (MPC) and meta-learning have been introduced to improve training efficiency and stability. Additionally, the emergence of deep reinforcement learning (DRL)—which integrates deep learning with reinforcement learning—continues to evolve, offering greater learning capacity and adaptability in dynamic environments [39].

##### Latest research trends in transfer learning

Transfer learning is a machine learning approach that transfers knowledge from a source task to a target task, offering particular advantages when training data is limited. This method significantly enhances both model accuracy and learning efficiency in data-scarce scenarios [40]. In recent years, researchers have widely applied transfer learning across various domains, including bioinformatics, drug target prediction, natural language processing, and computer vision [41–46]. For instance, in medical image analysis, transfer learning enables the adaptation of pre-trained models—originally trained on large-scale natural image datasets—for specialized tasks such as medical image classification, yielding strong performance outcomes [47, 48].

Current research in transfer learning focuses on several key challenges. First, researchers aim to identify optimal source and target task pairings to maximize the benefits of knowledge transfer. Second, they work to design robust transfer learning frameworks that mitigate the risk of negative transfer, which can degrade model performance. Third, integrating domain adaptation techniques remain essential for addressing distributional differences between source and target datasets [48]. In parallel, investigators are exploring hybrid strategies that combine transfer learning with deep learning and meta-learning to further boost model adaptability and performance.

#### Innovation in deep learning

Deep learning, a core discipline within artificial intelligence, has advanced rapidly in recent years. These developments center on the continual optimization of neural network architectures and the broadening of their application across diverse domains. Notably, innovations in convolutional neural networks (CNNs), recurrent neural networks (RNNs), and generative adversarial networks (GANs) have driven significant breakthroughs [49]. These advancements have not only improved model performance but also accelerated the intelligent transformation of the medical industry.

##### Advances in convolutional neural networks

CNNs have achieved remarkable success in image processing and computer vision tasks. By leveraging local perception and weight sharing, CNNs effectively extract spatial features from images, excelling in tasks such as image classification and object detection [50]. Recent innovations have led to the development of advanced CNN architectures, including ResNet and DenseNet. These networks significantly enhance depth and performance by incorporating residual and dense connections [51, 52]. Additionally, the introduction of new activation functions, such as the LogRelu activation function, and optimization algorithms like the Adam optimizer, has improved training efficiency and convergence [53].

CNNs have shown particular promise in medical image analysis. For instance, in lung cancer CT imaging, CNNs are employed to automatically detect and classify pulmonary nodules. Research has demonstrated that CNNs substantially improve diagnostic accuracy [54]. Furthermore, CNNs are applied in motion correction for MRI images, using Conditional Generative Adversarial Networks (CGANs) to predict brain images free from motion artifacts, thereby enhancing image quality [55]. These advancements not only streamline image processing but also provide more reliable support for clinical decision-making.

##### Application of recurrent neural networks with time series data

RNNs have garnered significant attention for their unique ability to process time series data. By maintaining hidden state connections across time steps, RNNs can retain information from previous inputs, making them highly effective for sequential data such as text, speech, and physiological signals [56, 57]. In medical applications, RNNs are widely used for patient monitoring and disease prediction. For instance, researchers have employed RNNs to predict the risk of heart attacks by analyzing patients’ physiological signal sequences [58–61].

Two prominent RNN variants, Long Short-Term Memory (LSTM) networks and Gated Recurrent Units (GRUs), address the challenges of vanishing and exploding gradients that hinder traditional RNNs in long-sequence learning [62]. These architectures introduce gating mechanisms that enable more stable and accurate performance when processing complex time series data. For example, when analyzing electrocardiogram (ECG) signals, LSTM networks effectively capture long-term dependencies in heart rate fluctuations, thereby enhancing early diagnosis of cardiovascular diseases [63–65].

Beyond healthcare, RNNs are also extensively applied in natural language processing (NLP) tasks, including sentiment analysis and machine translation [66, 67]. By modeling the sequential nature of text, RNNs capture contextual dependencies, significantly improving the accuracy and fluency of language understanding [68, 69].

##### Theory and application of generative adversarial networks

GANs have emerged as a major innovation in deep learning, primarily used to generate synthetic samples that closely resemble real training data [70]. A GAN comprises two competing neural networks—a generator and a discriminator—that are trained simultaneously through adversarial learning [71, 72]. The generator aims to produce realistic samples, while the discriminator attempts to distinguish between real and generated data. This adversarial process enables GANs to excel in various tasks, including image generation, restoration, and data augmentation [72–74].

In the medical domain, GANs have demonstrated substantial utility in synthesizing and enhancing medical images. For example, GANs can generate high-quality medical images to support the training of other deep learning models, particularly in data-scarce environments [75]. Studies show that GANs perform exceptionally well in generating cardiac MRI images, significantly improving image quality and diagnostic accuracy [76]. Additionally, researchers have used GANs to create synthetic electronic health records (EHRs), enabling model training and data analysis without compromising real patient data [77, 78].

Despite their advantages, GANs face several technical challenges, including unstable training dynamics and mode collapse. To address these issues, researchers have proposed various enhancements, such as introducing symmetry in adversarial training and refining loss functions to stabilize learning and improve sample diversity [79]. These advances continue to expand the potential applications of GANs, particularly in fields that demand high-quality synthetic data.

#### Breakthroughs in natural language processing

NLP, a core discipline within artificial intelligence, has advanced rapidly in recent years. Its applications span a wide range of fields, including text analysis, speech recognition, and machine translation. Driven by developments in deep learning and big data, NLP capabilities have significantly improved, particularly in language modeling, sentiment analysis, and real-time speech-to-text systems, thereby accelerating digital transformation across various industries [80].

##### Evolution of language models

The evolution of language models has marked a shift from traditional rule-based systems to powerful deep learning frameworks. Early models relied on statistical approaches, such as n-gram models, which predict the next word in a sequence based on word co-occurrence frequencies [81]. However, these models struggle with capturing long-range dependencies in text [82].

Recent breakthroughs in deep learning have dramatically enhanced language modeling. Architectures based on RNNs and, more notably, Transformers, have set new benchmarks in performance [83, 84]. Transformer models leverage self-attention mechanisms to efficiently capture global contextual information, enabling more accurate text comprehension and generation [85, 86]. Among these, Bidirectional Encoder Representations from Transformers (BERT) has achieved notable success by applying bidirectional training to understand the context surrounding each word, significantly improving performance in a wide range of NLP tasks [87, 88]. Similarly, the Generative Pre-trained Transformer (GPT) series employs large-scale unsupervised pre-training followed by fine-tuning on specific downstream tasks, yielding state-of-the-art results [89].

The success of these models has not only propelled the field of NLP forward but has also enabled practical innovations in intelligent dialogue systems, automated text generation, information retrieval, and predictive text analytics [90–96].

##### Latest achievements in speech recognition and sentiment analysis

Advances in speech recognition technology have enabled computers to accurately interpret and process human speech. Traditional systems relied heavily on manual feature extraction and pattern matching, whereas modern approaches primarily adopt deep learning architectures such as CNNs and LSTMs [97]. These models automatically learn meaningful feature representations directly from raw audio signals, significantly enhancing both the accuracy and speed of recognition tasks [98–104].

In sentiment analysis, NLP allows machines to detect and interpret emotional tone and attitudes in text. This capability is particularly critical in serious illness communication, where effective care requires a nuanced understanding of patients' values and beliefs about end-of-life treatment. NLP offers a scalable and objective method to quantify value- and belief-oriented content in real-world clinical conversations [105]. Additionally, analyzing text from social media, patient feedback, and online discussions provides valuable insights into patients’ emotional states and evolving needs in real time [106–109].

For instance, researchers have used sentiment lexicons and deep learning models to extract emotional signals from large-scale textual data, contributing to disease surveillance and offering targeted psychological support [110, 111]. These findings underscore the critical role of sentiment analysis in both mental health monitoring and public opinion assessment.

##### Progress and challenges in machine translation

Advancements in machine translation have significantly improved cross-linguistic communication. Traditional rule-based translation methods have largely been supplanted by neural machine translation (NMT) systems, which leverage deep learning to generate more fluent and natural translations [112–114]. By training on large-scale bilingual parallel corpora, NMT models learn to capture complex syntactic and semantic patterns. In particular, the introduction of the Transformer architecture has greatly enhanced translation performance, especially for long sentences and syntactically complex structures [115–118]. Despite these advances, NMT still faces several persistent challenges [119–121]. The inherent diversity and structural complexity of natural languages often lead to inaccuracies in domain-specific terminology and idiomatic expressions. Moreover, translating low-resource languages remains difficult due to the limited availability of annotated data. To address these issues, researchers are developing novel strategies such as multimodal learning and transfer learning, which aim to improve translation quality and adaptability across varied linguistic contexts [122–125]. In summary, breakthroughs in NLP -particularly in machine translation—have profoundly influenced numerous industries, including healthcare. As these technologies continue to evolve and their applications deepen, we expect NLP to further enhance the intelligence, accessibility, and efficiency of human–computer interaction.

#### Developments in computer vision

Computer vision is a pivotal subfield of artificial intelligence that focuses on enabling computers to interpret and process visual data. With the rapid advancements in deep learning and big data technologies, computer vision has seen expanding applications, particularly in the medical domain [126–128]. Recent progress in computer vision has not only increased the efficiency of image processing but also driven the digital transformation of related industries. The evolution of technologies such as image recognition, object detection, and image segmentation has laid a robust foundation for ongoing developments in the field.

##### Current application statuses in image recognition technology

Image recognition is a central task in computer vision, designed to extract meaningful information from images for classification purposes. Substantial progress has been made in various fields, particularly in healthcare. In the medical sector, image recognition techniques are extensively applied to assist in disease diagnosis, offering doctors faster and more accurate results [129]. Research has demonstrated that deep learning-based medical image recognition models outperform traditional methods, especially in tasks such as tumor detection and pathological image analysis, where these technologies are now widely used [130].

Despite these advancements, challenges persist in image recognition technology [131–136]. First, the diversity and complexity of image data hinder the generalization capabilities of algorithms. Additionally, factors such as lighting conditions, viewing angles, and background noise often reduce the accuracy of recognition systems. As a result, improving robustness and precision of image recognition continues to be a major focus of research.

##### New methods for object detection and image segmentation

Object detection and image segmentation are two fundamental tasks in computer vision. Object detection focuses on identifying specific objects within an image and determining their locations, while image segmentation involves partitioning the image into multiple meaningful regions [137–140]. With the advancement of deep learning, both tasks have undergone significant innovation and achieved remarkable performance gains in recent years [141–145].

For object detection, CNN-based algorithms have become the dominant approach. Popular models include You Only Look Once (YOLO), Faster R-CNN, R-CNN, and Fast R-CNN [146–149]. These methods employ end-to-end training strategies to balance real-time processing with high detection accuracy. For instance, Faster R-CNN enhances both speed and accuracy by integrating the Region Proposal Network (RPN) [150–152]. To further improve the detection of small objects, researchers have introduced multi-scale feature fusion techniques, which enhance performance across varying object sizes [153–156].

In image segmentation, deep learning models such as U-Net and Mask R-CNN have shown outstanding results in both medical and natural image segmentation tasks [157–161]. U-Net, in particular, excels in medical image segmentation due to its symmetric encoder-decoder architecture, which effectively captures contextual information and enables high-precision segmentation [162]. Mask R-CNN demonstrates superior performance in instance segmentation by simultaneously performing object detection and segmentation, making it highly effective for multi-object recognition in complex environments [163, 164].

Despite these advances, object detection and image segmentation still face key challenges. Handling occlusions in cluttered backgrounds and improving model adaptability in dynamic or variable scenes remain critical areas for future research.

### Application of AI in depression

#### Application of AI in sentiment analysis

##### Natural language processing

NLP, a critical branch of artificial intelligence, focuses on enabling computers to understand, interpret, and generate human language. In recent years, NLP has seen increasing application in sentiment analysis, particularly in research on depression [165, 166]. By analyzing textual data, NLP can detect emotional states, especially negative emotions such as depression and anxiety [167–170]. Studies have demonstrated that NLP techniques can effectively extract emotional signals from sources such as social media, online comments, and other text-based platforms, thereby supporting mental health interventions [171–174]. For instance, deep learning-based sentiment analysis of social media content can track emotional fluctuations in individuals with depression, allowing for timely and targeted interventions [175–177].

Moreover, NLP can identify potential mental health concerns by analyzing patients’ linguistic patterns. Research has shown that individuals with depression often exhibit distinct emotional markers in their language, including frequent use of negative words and expressions of hopelessness. NLP can quantify and interpret these patterns [177–179]. This approach not only improves the precision of sentiment analysis but also provides a robust data foundation for personalized psychological interventions.

##### Mining of social media data

Social media platforms such as Twitter and Facebook have become valuable data sources for sentiment analysis. User-generated content on these platforms contains rich emotional information that often reflects individuals’ psychological states. By mining social media data, researchers can monitor changes in public attitudes toward depression and track emotional responses over time. For example, during the COVID-19 pandemic, online discussions surrounding mental health surged. Studies have shown that social media data can reliably capture fluctuations in public sentiment and provide real-time insights for guiding mental health interventions [180–184].

In addition, analyzing social media content can reveal emotional patterns specific to individuals with depression. Research indicates that people with depression tend to exhibit higher levels of negative emotional expression online, often closely linked to their mental health status [185–187]. By examining these data, researchers can identify high-risk individuals who may require psychological support, thereby enabling more targeted and timelier mental health services.

##### Real-time monitoring of emotional changes

Real-time monitoring of emotional changes represents a critical application of AI in sentiment analysis. Researchers can leverage wearable devices and mobile applications to collect both physiological data and self-reported emotional feedback, enabling continuous assessment of an individual’s emotional state [188, 189]. This approach offers the advantage of capturing moment-to-moment emotional fluctuations, supporting dynamic analysis and facilitating early psychological intervention [190]. For instance, studies have shown that monitoring user activity levels, sleep patterns, and social interactions can effectively predict emotional changes and help detect emerging psychological crises [191].

Furthermore, combining NLP with real-time physiological monitoring enables a more comprehensive understanding of emotional dynamics. By analyzing social media posts alongside physiological data, researchers can detect patterns of emotional fluctuation and deliver personalized psychological intervention strategies based on those patterns [192, 193]. This integrated approach enhances the accuracy of emotional state detection and offers more targeted support for individuals with depression.

#### Role of AI in early diagnosis of depression

##### Integration of machine learning algorithms

Machine learning has played a critical role in advancing early depression diagnosis. Traditional diagnostic approaches often depend on clinicians' subjective judgment, which may lead to misdiagnosis or missed cases. With the rise of artificial intelligence, researchers have increasingly applied machine learning algorithms to depression diagnosis research [194]. These algorithms can process large volumes of patient data, identify hidden patterns and features, and thereby enhance diagnostic accuracy. For example, studies have shown that machine learning models can predict depression risk by analyzing physiological data, psychological assessments, and socioeconomic factors [195]. By integrating multiple algorithms, researchers can leverage the strengths of different models to further improve diagnostic precision and reliability [196, 197].

Early-generation diagnostic methods primarily relied on knowledge-driven feature selection. While effective in structured scenarios, these methods often failed to capture complex nonlinear relationships in clinical data. Second-generation approaches shifted to data-driven learning, enabling automatic feature extraction. However, they typically require large, high-quality datasets and often suffer from limited interpretability. To overcome these limitations, researchers have introduced third-generation hybrid approaches that integrate both knowledge- and data-driven techniques. These models significantly improve diagnostic accuracy yet still face challenges in interpretability [198]. Moving forward, it is essential to refine algorithm fusion strategies and enhance model transparency to better support early and reliable depression diagnosis.

##### Combination of biomarkers and AI

Biomarkers—specific molecules found in blood, saliva, or other biological samples—can reflect an individual's physiological state and mental health status [199, 200]. For example, studies have identified inflammatory markers such as GlycA as being strongly associated with both the onset and persistence of depression, offering a promising direction for biomarker research in this domain [201]. By combining biomarker data with machine learning algorithms, researchers can build highly accurate predictive models that not only detect depression but also forecast its progression.

Machine learning algorithms can extract meaningful patterns from high-dimensional biological data, enabling more informed clinical decisions. Several studies have demonstrated that models combining biomarkers with AI can effectively distinguish individuals with mental health disorders, including depression, from healthy controls—with reported prediction accuracies reaching up to 99% [202–204]. This integration not only enhances early detection but also supports the development of personalized treatment strategies tailored to individual patients.

##### Establishment and verification of prediction models

Building and validating predictive models is a critical component of applying AI in the early diagnosis of depression. Researchers often develop comprehensive models by integrating diverse data sources, including clinical assessments, psychological evaluations, and biomarkers. These models are typically trained using machine learning algorithms to identify key factors associated with the onset of depression. For example, one study used national health survey data and machine learning techniques to construct a predictive model capable of forecasting future depression with an accuracy of 87% [205]. In another study, researchers combined clinical psychological assessments with biomarkers to develop models that predict the likelihood of sustained remission and treatment outcomes for depression [206].

To ensure the generalizability and reliability of these models, researchers employ validation techniques such as cross-validation and external validation. These methods test the model on independent datasets to evaluate its performance in real-world scenarios. For instance, comparative analyses of different machine learning algorithms have shown that random forest models often outperform other approaches in predicting depression, with reported AUC values reaching 0.91 [207]. Such validation efforts not only strengthen the credibility of predictive models but also lay the theoretical groundwork for their clinical application.

#### Formulation of personalized treatment plans

##### Data-driven treatment strategies

The development of personalized treatment plans, increasingly driven by AI, plays a crucial role in the management of depression. By analyzing extensive clinical data and patient-specific information, data-driven treatment strategies enable the design of more precise, individualized care plans. For example, research demonstrates that AI can assist clinicians in selecting the most appropriate treatment by analyzing a patient’s genomic data, lifestyle, and psychological profile [208]. Through information fusion, not only can the therapeutic effect be improved, but also the interpretability of the diagnosis can be enhanced [195]. Moreover, these data-driven strategies facilitate dynamic, personalized care by continuously monitoring patient responses in real-time and promptly adjusting treatment plans as needed [198, 209].

AI has already shown promise in improving the treatment of depression. Through machine learning algorithms, researchers can identify varying treatment responses among patients and develop tailored therapeutic approaches based on these responses. For instance, while some patients may benefit from SSRIs, others may respond more favorably to alternative medications [210–213]. This personalized treatment approach not only enhances therapeutic outcomes but also provides patients with a more compassionate and individualized healthcare experience.

##### Application of feedback mechanism

The feedback mechanism is integral to the development of personalized treatment plans. By collecting and analyzing real-time data on patients' treatment responses, clinicians can promptly adjust treatment strategies based on patient feedback. For instance, patients can use mobile health applications to record emotional fluctuations and treatment outcomes. AI algorithms can then analyze this data, providing valuable decision support for clinicians [214, 215]. This feedback loop not only enhances patient engagement but also improves the relevance and effectiveness of the treatment.

Furthermore, the feedback mechanism fosters better self-management skills among patients. Studies indicate that when patients receive timely feedback on their condition and treatment efficacy, they typically exhibit higher compliance and a more positive outlook on their treatment [216–218]. This feedback is applicable not only to pharmacological treatments but also to other therapeutic approaches, such as psychotherapy and behavioral interventions. By establishing an effective feedback system, healthcare teams can more accurately assess treatment progress and make necessary adjustments, thereby optimizing overall treatment outcomes.

##### Evaluation of individualized treatment effects

Evaluating the effects of individualized treatment is crucial to ensuring the success of treatment plans. By systematically assessing patients' responses, clinicians can determine the effectiveness of various treatment strategies, providing valuable insights for future decision-making. In the treatment of depression, the selection of appropriate assessment tools is essential. Commonly used instruments include Self-Rating Depression Scales, such as the PHQ-9, and clinical assessment scales, such as the HAMD. These tools help clinicians quantify changes in patient symptoms and track treatment progress [219–222].

With the advancement of AI, there is growing interest in using machine learning and data analysis techniques to evaluate therapeutic outcomes. For example, by analyzing patients' electronic health records (EHR), researchers can identify the factors most influencing treatment effectiveness and optimize treatment plans accordingly [223–225]. This data-driven approach not only improves assessment accuracy but also strengthens the foundation for personalized care.

It is also crucial to consider patients' subjective experiences when evaluating treatment effects. Studies show that patients' satisfaction with their treatment and their improved quality of life are closely linked to treatment success [226, 227]. Therefore, when assessing therapeutic outcomes, clinicians should consider not only quantitative clinical indicators but also patients' subjective feedback. By taking these factors into account, healthcare teams can more comprehensively assess the effectiveness of individualized treatments, thereby providing enhanced care for patients.

### Application of AI in target prediction for antidepressant Chinese medicine

AI is becoming increasingly integral to the drug discovery process. It aids researchers in identifying potential drug targets and candidate compounds by analyzing complex biological and chemical data. For instance, machine learning algorithms can extract valuable insights from vast biological datasets, predict the activity and toxicity of compounds, and expedite the drug screening process [228, 229]. Furthermore, AI contributes to drug design by generating new molecular structures and optimizing their properties, thus enhancing the likelihood of successful drug development [230]. AI has significantly shortened drug development cycles, reduced research and development costs, and improved the success rates of new drug approvals [231].

#### Current status of research on antidepressant Chinese medicine targets

##### Multi-component and multi-target characteristics of chinese medicine

Chinese medicine, with its multi-component and multi-target characteristics, offers unique advantages in treating complex diseases such as depression. Typically, Chinese medicine consists of various plant components that interact through diverse biological pathways to exert a synergistic effect. For example, certain components in Chinese medicine can produce antidepressant effects by regulating neurotransmitter balances, influencing the endocrine system, and modulating immune responses [232, 233]. This multi-target approach contrasts with the single-target strategy commonly used in modern drug development, which often encounters issues such as drug tolerance and side effects. By leveraging the multi-component and multi-target nature of Chinese medicine, researchers can explore new avenues for depression treatment, offering innovative strategies for managing the disorder.

##### Limitations of traditional target discovery methods

Traditional target discovery methods primarily focus on validating single targets and screening potential drugs, often employing techniques such as high-throughput screening (HTS) and in vitro experiments [234, 235]. While these methods can identify potential drug targets to some extent, they are limited in their ability to fully capture the complexities of diseases like depression, which involve multiple biological pathways and intricate interaction networks. Consequently, studies centered on single targets often fail to accurately reflect the overall therapeutic effects of drugs [236–238]. In addition, traditional approaches are constrained by significant time and cost limitations, hindering their capacity to meet the demands of modern drug discovery. Therefore, there is a pressing need to explore more efficient methods for target discovery, such as AI-driven technologies, to comprehensively analyze Chinese medicine compound prescriptions and systematically identify novel drug targets.

##### Research on antidepressant Chinese medicine targets

Recent research on the targets of antidepressant Chinese medicines has significantly increased. Emerging technologies, such as network pharmacology, have allowed researchers to uncover the multi-target mechanisms underlying the effects of Chinese medicine components. For example, studies have demonstrated that certain components of Chinese medicine alleviate depressive symptoms by regulating neurotransmitters (*e.g*., 5-HT, NE) and inflammatory markers [239, 240]. Additionally, modern biotechnologies have enabled the identification of interactions between Chinese medicine components and target proteins, providing a solid theoretical foundation for their clinical application [238]. From this analysis, it is clear that the research on antidepressant Chinese medicines is increasingly focused on multiple components and targets. The limitations of traditional target discovery methods have driven the exploration of innovative technologies.

#### Application of AI in the discovery of antidepressant Chinese medicine targets

##### Application of machine learning algorithms

The application of machine learning algorithms in discovering antidepressant targets in Chinese medicine has garnered increasing attention, particularly in managing complex biomedical data. By analyzing various Chinese medicine components and their corresponding targets, machine learning can efficiently identify potential drug targets. For example, the TCMBank database offers comprehensive data on 9,192 medicinal materials, 61,966 components, and 15,179 targets, which forms a solid foundation for applying machine learning algorithms in target discovery [241]. Leveraging these data with deep learning models, researchers have successfully identified potential targets related to depression.

In one specific study, researchers employed machine learning algorithms to analyze the components of *Hypericum* perforatum and identified key components—such as quercetin, kaempferol, and luteolin—that are associated with depression-related targets. These components demonstrated strong binding affinity, interacting with five key targets: AKT1, MAPK1, MYC, EGF, and HSP90AA1 [242]. Constructing component-target networks allows for a more intuitive understanding of the mechanisms through which Chinese medicine components exert their effects, providing valuable data for the development of antidepressant drugs.

Moreover, machine learning can also aid in identifying biomarkers for depression by analyzing patient data, facilitating the advancement of personalized treatment strategies. For instance, AI can uncover genetic variations associated with depression by analyzing genomic data from patients, offering new insights for target discovery in Chinese medicine [243–245]. This integration of AI enhances the efficiency of target discovery and paves the way for modernizing Chinese medicine.

##### Progress and application of data mining technology

Recent advances in data mining technologies have significantly accelerated the identification of therapeutic targets for antidepressant Chinese medicine. By systematically analyzing the chemical components of Chinese medicinal formulations and their corresponding biological targets, data mining enables the elucidation of potential mechanisms underlying drug action. For example, in-depth mining of the TCMBank database has uncovered numerous bioactive compounds and their associated targets linked to depression, enabling the construction of comprehensive component–target–disease network models. These models enhance our understanding of how Chinese medicine exerts its effects and offer critical insights for novel drug development [241].

In practical applications, data mining can integrate gene expression and clinical datasets to pinpoint key depression-related genes. Several studies have identified differentially expressed genes associated with specific Chinese medicine components by analyzing the gene expression profiles of patients with depression. These genes may serve as promising new targets for antidepressant therapy [246, 247]. Additionally, mining the scientific literature allows researchers to systematically assess the pharmacological relevance of traditional medicine, revealing correlations between specific compounds and depression that support target discovery.

As data mining continues to evolve, advanced methods that integrate machine learning and deep learning have begun to transform the target discovery process in Chinese medicine. Algorithms such as Support Vector Machines (SVM) and Random Forest can extract actionable insights from high-dimensional biological data, enabling the identification of novel therapeutic targets with greater efficiency and precision [248]. The integration of these computational approaches is reshaping the landscape of drug discovery in Chinese medicine and advancing the development of more effective antidepressant therapies.

##### Integration of AI and Chinese medicine databases

Integrating AI with databases of Chinese medicine components offers powerful support for identifying targets of antidepressant therapies derived from traditional medicine. Comprehensive databases such as TCMBank and other related repositories contain extensive information on chemical constituents, molecular targets, and associated diseases, forming a critical foundation for AI-driven analyses. By deeply interrogating these resources, AI can rapidly identify bioactive compounds linked to depression and elucidate their underlying mechanisms of action.

For example, the development of the TCMBank database has enabled large-scale AI-assisted data mining to pinpoint depression-related components and targets. This database not only includes detailed chemical structure information but also catalogs the interactions between compounds and biological targets. Using these data, researchers can construct component–target networks that shed light on the molecular mechanisms of traditional Chinese medicine [241]. This data-driven framework brings greater scientific rigor and systematicity to target discovery in the context of Chinese medicine.

Moreover, AI can apply NLP to extract valuable insights from the scientific literature, further enriching existing databases. For instance, researchers can employ AI to automatically retrieve information about depression-associated compounds and targets from published studies, providing additional leads for therapeutic target identification [245].

##### Integration of AI and experimental verification

With the rapid advancement of AI, an increasing number of studies now emphasize integrating AI-based predictions with experimental validation to enhance the reliability and biological relevance of results. In the field of antidepressant drug discovery from traditional Chinese medicine, AI models can rapidly analyze large-scale biological datasets to identify potential therapeutic targets. However, predictions generated solely by AI are not yet suitable for direct clinical application and must be experimentally validated to confirm their biological significance. For example, researchers used a deep learning model to predict the antidepressant effects of various traditional Chinese medicine components. They subsequently verified the biological activity of the predicted targets through cell-based experiments. The results demonstrated a strong concordance between the AI-generated predictions and experimental findings, reinforcing the utility of AI in accelerating target discovery [242]. This case exemplifies the essential role of experimental validation in confirming AI-derived predictions and underscores the transformative potential of AI in drug development.

Experimental data play a critical role in validating AI-generated predictions and serve as a foundation for iterative model refinement. By comparing experimental outcomes with AI predictions, researchers can identify limitations in model performance and make targeted adjustments to improve accuracy. Specifically, collecting large-scale experimental datasets—such as drug bioactivity profiles and target expression levels—allows researchers to feed empirical evidence back into the AI model for optimization [249]. Incorporating these data helps the model determine which input features most strongly influence target prediction, enabling more precise feature selection and fine-tuning of model parameters. This feedback-driven optimization enhances both the predictive reliability and practical applicability of the model. Ultimately, this iterative refinement process provides a robust framework for advancing the discovery of antidepressant targets in Chinese medicine and opens new avenues for data-informed therapeutic development.

#### Future development directions

##### Continuous AI progress and emergence of new algorithms

In recent years, AI has moved beyond basic research and begun to influence clinical practice in the field of depression, with notable impact in drug discovery and target identification [250, 251]. As computational power continues to increase and data become more readily accessible, we anticipate the emergence of more sophisticated algorithms capable of handling complex, high-dimensional datasets. These innovations will likely improve both the accuracy and efficiency of target discovery. For example, cutting-edge technologies such as GANs and reinforcement learning may play critical roles in future drug screening and target validation efforts [20, 252]. In parallel, ongoing progress in AI is expected to accelerate the development of personalized medicine. We envision that future AI-driven systems will offer highly tailored therapeutic recommendations—identifying optimal drug targets and treatment strategies based on a patient’s clinical data, diagnostic results, and lifestyle factors.

##### Standardization and normalization of target discovery in chinese medicine

The process of discovering targets in Chinese medicine faces significant challenges related to standardization and normalization. These issues not only hinder the reproducibility of research but also limit the integration of Chinese medicine into modern medical practices. Future efforts should focus on establishing unified standards and guidelines to direct the research and application of Chinese medicine targets. This can be achieved by developing clear research protocols and standardized data collection and analysis methods, ensuring consistency across different research teams involved in target discovery. By implementing standardized methodologies and normalized research practices, the modernization and internationalization of Chinese medicine will be better supported, facilitating its broader acceptance and application worldwide.

##### Need for interdisciplinary cooperation and multi-field integration

Interdisciplinary collaboration plays a vital role in advancing target discovery in Chinese medicine. This field inherently spans multiple disciplines—including pharmacology, chemistry, biology, and computer science—making cross-disciplinary communication and cooperation essential for accelerating progress. For example, pharmacologists can partner with computer scientists to harness AI for analyzing large-scale biological datasets and identifying potential therapeutic targets. Moreover, interdisciplinary integration fosters technological innovation and expands the scope of research applications. By combining bioinformatics and systems biology approaches, researchers can gain a deeper understanding of the mechanisms of action and complex target networks associated with Chinese medicine. Looking ahead, establishing collaborative research platforms and promoting active engagement among experts across diverse fields will be key to driving future advancements in target discovery.

## Application of omics in research for antidepressant Chinese medicine targets

### Overview of omics technology

**Fig. 1:**
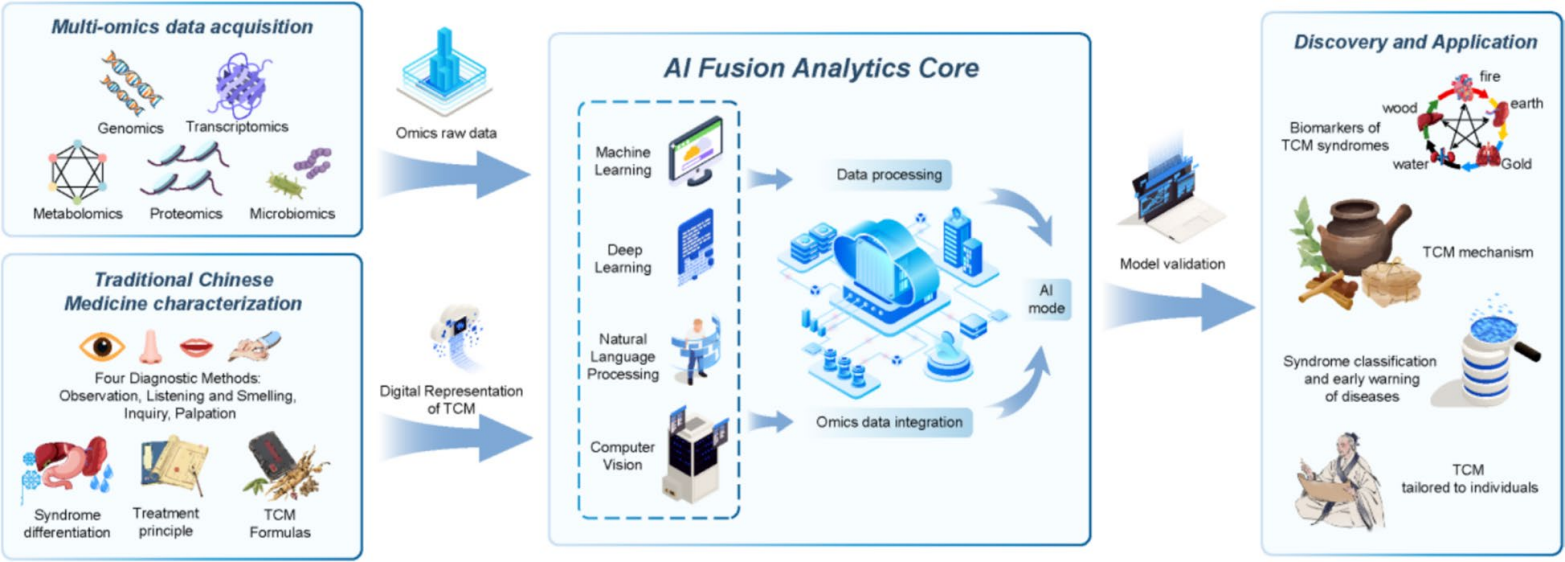
Schematic diagram of AI and omics in traditional Chinese medicine research. AI: As a powerful "engine" and "brain", it is responsible for processing and understanding massive and complex data. Multi-omics: As an advanced "microscope" and "detector", it reveals the inner details of life at various levels. Traditional Chinese medicine theory: As the "navigation map" and "soul" of the whole, it provides the framework, direction and ultimate goal of research

Omics technology is a cornerstone of modern biomedical research, playing a critical role in the study of antidepressant Chinese medicine. By employing high-throughput analytical techniques, omics technologies enable researchers to gain a comprehensive understanding of molecular processes within organisms, thereby accelerating the identification and development of drug targets. As technological advancements continue, the application of omics technologies in drug target research expands, offering new insights that are pivotal for precision medicine and personalized treatment strategies [253–255]. The illustration of AI and omics in traditional Chinese medicine research is shown in Fig. 1.

#### Genomics

Genomics is the study of an organism's genome structure and function, and its relationship to phenotype. It focuses on gene expression levels and how these change under different physiological or pathological conditions. High-throughput sequencing technologies allow researchers to rapidly obtain genomic data from individuals, facilitating the identification of genetic variations associated with diseases or drug responses [256, 257]. For instance, genome-wide association studies (GWAS) enable scientists to uncover genetic variations linked to specific diseases, providing a foundation for the development of targeted therapies [258, 259]. Moreover, genomics not only aids in identifying potential drug targets but also offers insights into drug mechanisms and the variability in individual responses to treatments [260].

The rapid advancement of genomic sequencing technologies has opened new frontiers in medical research. Since the completion of the Human Genome Project, sequencing methods have undergone dramatic improvements, transitioning from early Sanger sequencing to today’s high-throughput next-generation sequencing (NGS) technologies. In particular, third-generation sequencing technologies, such as Oxford Nanopore and PacBio, have significantly lowered sequencing costs, accelerated data processing, and enhanced accuracy [261]. These technologies not only generate complete genomic sequences rapidly but also identify structural variations and repetitive sequences within complex genomes. This capability significantly advances the development of personalized and precision medicine [262]. The long read lengths characteristic of third-generation sequencing technologies enable researchers to resolve complex genomic structures more effectively, particularly excelling in animal genome assembly. These methods yield higher-quality genome assemblies, which reveal both the evolutionary history and functional attributes of the genome [263].

Single-cell genomics, a groundbreaking technology developed in recent years, allows for the study of genomic heterogeneity and complexity at the individual cell level. Unlike traditional population-based cell sequencing, single-cell genomics provides detailed insights into gene expression profiles and genetic variations across different cell types, preserving crucial cell-specific information [264, 265]. This technology has numerous applications, such as examining molecular changes in nervous system cells and classifying cell types [266, 267]. The primary advantage of single-cell genomics lies in its ability to uncover intercellular differences, which is essential for understanding disease development mechanisms [268].

#### Transcriptomics

Transcriptomics is a technique used to study RNA molecules within cells, particularly focusing on mRNA expression. By analyzing gene expression patterns under various conditions, researchers can infer the roles of genes in biological processes. In drug target discovery, transcriptomics helps identify genes that are upregulated or downregulated in disease states, providing potential targets for targeted therapies. It also offers insights into the biological functions and signaling pathways associated with these genes. Additionally, transcriptomic analysis enables the identification of disease-related biomarkers, which can be used for diagnostic monitoring and treatment, thereby supporting personalized medicine [269].

The rapid advancement of RNA sequencing (RNA-Seq) technology has significantly enhanced transcriptomic research. Compared to traditional microarray techniques, RNA-Seq offers superior sensitivity, resolution, and the ability to simultaneously detect the expression levels of thousands of genes. Moreover, the advent of single-cell RNA sequencing technology has enabled researchers to investigate gene expression heterogeneity at the single-cell level, providing a novel perspective on cellular differences and their roles in disease [264, 270].

#### Metabolomics

Metabolomics is the study of small molecule metabolites in living organisms, focusing on their composition and changes. This field provides insights into the metabolic state of cells and their interactions with the environment, helping to uncover biological processes and disease mechanisms. Recently, metabolomics has become a valuable tool in disease diagnosis and drug target evaluation [271]. For example, metabolomics can identify biomarkers associated with specific diseases, aiding in early diagnosis and monitoring disease progression [272, 273].

Key metabolomics techniques include mass spectrometry (MS), nuclear magnetic resonance (NMR), liquid chromatography (LC), and gas chromatography (GC). These methods offer high sensitivity and resolution, enabling detailed analysis of metabolites and enhancing our understanding of cellular metabolic pathways and their roles in health and disease [274].

#### Proteomics

Proteomics investigates the composition, structure, and function of proteins in living organisms. By analyzing the proteome, researchers can identify disease-associated biomarkers and elucidate their roles in disease onset and progression. Additionally, proteomics holds significant promise for applications in drug development and toxicological assessment [275–277].

In recent years, mass spectrometry-based quantification methods—both label-based and label-free—have become essential tools in proteomics. These approaches enable the simultaneous quantification of thousands of proteins in a single experiment, substantially improving both research throughput and data robustness. For instance, the Tandem Mass Tag (TMT) labeling technique allows direct comparison of protein expression across multiple samples, facilitating the identification of differentially expressed proteins. This capability is critical for uncovering disease mechanisms and discovering novel biomarkers [278, 279].

#### Microbiomics

Microbiomics, a rapidly growing field, has garnered significant attention in recent years. Advances in high-throughput sequencing and bioinformatics have provided deeper insights into the composition and function of the microbiome, as well as its interactions with host health and disease. The microbiome plays a crucial role in maintaining the host’s physiological balance and is closely linked to the onset and progression of various diseases [280–282].

The human microbiome consists of microorganisms, including bacteria, fungi, viruses, and archaea, that reside within our bodies. These microbes are primarily found in the gut, skin, mouth, and respiratory tract. Research has demonstrated that the gut microbiome is essential for digestion, metabolism, immune regulation, and neural signaling. The composition and function of the microbiome are intricately tied to the host’s health. The gut microbiota aids in breaking down complex food components, synthesizing vitamins and short-chain fatty acids, and regulating immune responses. Additionally, it significantly influences neurological conditions such as depression and anxiety. Evidence suggests that the microbiome’s composition and function are directly linked to the host’s overall health. Notably, the gut microbiome may impact mood and cognitive function via the gut-brain axis [283–285].

#### Rise of spatial omics

Recent advances in this field have enabled researchers to decipher the intricate architecture and functions of tissues at single-cell resolution, surpassing the capabilities of traditional omics techniques. At the core of spatial omics lies the integration of high-resolution imaging with molecular profiling, allowing the simultaneous detection of multiple biomolecular expressions within a single tissue section [286]. This approach not only uncovers intercellular interactions but also provides detailed insights into the cellular microenvironment, offering a powerful framework for exploring complex biological processes and disease mechanisms [287]. The rapid evolution of spatial omics has been driven by progress in imaging technologies—such as fluorescence imaging and mass spectrometry imaging—and in computational analysis, particularly machine learning and bioinformatics. Together, these advances enable high-dimensional molecular characterization at the tissue level [288]. In recent years, continued improvements in sequencing and imaging technologies have broadened the scope of spatial omics. Initially focused on genomics and transcriptomics, the field now extends into proteomics and metabolomics, greatly expanding its utility across diverse areas of biomedical research [289].

The origins of spatial omics trace back to early tissue section analyses, which primarily relied on microscopy and immunohistochemistry. These traditional techniques provided limited spatial resolution and insufficient molecular detail. The advent of high-throughput sequencing prompted researchers to integrate spatial context with molecular profiling, leading to the emergence of spatial transcriptomics, spatial proteomics, and spatial metabolomics. In recent years, novel methods such as Spatial Transcriptomics and Spatial Mass Spectrometry Imaging have enabled high-resolution molecular imaging within tissue sections. These technologies have significantly accelerated biomedical research by offering unprecedented insights into tissue architecture and molecular heterogeneity [290, 291].

### Application of omics in depression

#### Application of genomics in depression

##### Identification of genes related to depression

Genomics plays a key role in depression research by enabling the identification of genes associated with the disorder. Recent advances in GWAS have uncovered numerous genetic variants linked to depression. For example, studies have identified genes such as brain-derived neurotrophic factor (BDNF) and the serotonin transporter gene (SLC6A4) as being closely associated with depression susceptibility [292–294]. Polymorphisms in these genes are believed to contribute significantly to the pathogenesis of depression. In addition, recent studies have identified novel candidate genes, including C4A, which is involved in synaptic pruning and may influence the neurobiological mechanisms underlying depression [295]. Investigating these genes in greater detail allows researchers to deepen their understanding of the genetic architecture of depression and to identify potential biomarkers for personalized treatment strategies. For instance, the expression levels of specific genes have been correlated with symptom severity, offering promising avenues for targeted clinical interventions [296].

##### Research on genetic susceptibility

Research on genetic susceptibility represents a critical dimension of genomics in the study of depression. Evidence strongly supports the heritability of depression, with twin studies estimating heritability at approximately 37–50% [297, 298]. Using polygenic risk scores (PRS), researchers can evaluate an individual’s genetic predisposition and estimate their risk of developing depression [299–301]. Recent findings indicate that genetic susceptibility influences not only the likelihood of developing depression but also the individual’s sensitivity to environmental stressors. For example, specific gene polymorphisms have been linked to variations in depressive symptoms in response to major life stress, underscoring the important role genetic factors play in the disorder’s pathogenesis [302, 303]. Moreover, studies have shown that interactions between genetic risk and socioeconomic conditions can further elevate depression risk. These findings highlight the need to consider both genetic and environmental influences when investigating the etiology of depression [304].

##### Interaction between genes and the environment

The interaction between genes and the environment is crucial for understanding the complex etiology of depression. Research demonstrates that environmental factors such as stress, life events, and social support can interact with an individual’s genetic makeup, influencing the onset and progression of depression. For example, variations in the FKBP5 gene have been shown to modulate an individual’s response to environmental stress, thereby affecting the severity of depressive symptoms [305].

Moreover, the impact of environmental factors can differ depending on an individual’s genetic background. Studies suggest that certain genetic variations may heighten the risk of depression in high-stress environments, while having little effect in low-stress settings [306]. This gene-environment interaction not only provides new insights for depression prevention and intervention but also forms the basis for developing personalized treatment strategies.

##### New research ideas for spatial genomics

Spatial genomics, an emerging field of research, provides deeper insights into the regulatory mechanisms of gene expression and its connection to disease states by analyzing the spatial organization of genomes [307]. This approach allows researchers to pinpoint the spatial localization of genes within the cell nucleus and examine their interactions with other genes [308, 309]. Such analysis is crucial for understanding the mechanisms underlying depression. By assessing gene expression across different brain regions, researchers can identify specific genes linked to depression, revealing their spatial specificity and variability. This highlights the biological foundation of depression. For instance, spatial transcriptomics has been employed to map gene expression patterns associated with depression, further exploring the spatial distribution of these genes within various cell types [310].

Studies have shown that the expression of the VRK2 gene is linked to depression risk, with its regulatory mechanisms demonstrating significant spatial specificity in affected individuals [311]. Additionally, emerging biomarkers like ZNF184 have been proposed as potential diagnostic and prognostic indicators for depression, with notable differential expression across distinct brain regions in patients [312]. As research progresses, spatial genomics is poised to uncover additional biomarkers associated with depression, advancing the field of precision medicine for this disorder. A summary of genomic studies related to depression is presented in Table 1.

**Table 1:** Characteristics of genomic studies in depression

Biomarkers	Analytical technique	Research objects/samples	Model	References
102 independent variants, 269 genes, and 15 genesets associated with depression	Genome-wide meta-analysis	807,553 individuals (246,363 cases and 561,190 controls)	/	[313]
rs4656484 at 1q24.1. Clinical depression risk was negatively genetically correlated with body mass index in individuals of East Asian descent	Genome-wide association studies (GWAS)	15,771 individuals with depression and 178,777 control individuals of East Asian descent	/	[314]
11 driver causal genes related to MDD symptoms (ESR1, RUNX1, PPARA, WWOX, etc.)	Genomics	Blood samples (297 patients)	/	[315]
17 independent loci that are significantly associated across the three phenotypes	GWAS	322,580 UK Biobank participants	/	[316]
hsa-miR-139-5p	GWAS	blood-derived exosomes from subjects	/	[317]
PSMB4	GWAS	807,553 individuals (246,363 cases and 561,190 controls)	/	[318]
Whole-genome single nucleotide variants (SNVs) distribution on the genomic regions may be related to major depression	whole-genome sequencing (WGS)	25 human subjects	/	[319]
Six identified methylation CpG sites	Genome-wide DNA methylation	Adult-onset depression (AOD; age ≥ 50 years, age at depression onset < 50 years; N = 10) and late-onset depression (LOD; age ≥ 50 years, age at depression onset ≥ 50 years; N = 25)	/	[320]
serotonin transporter gene (SLC6A4)	DNA methylation	146 inpatients from the database of the Ulm Gene Brain Behavior Project (UGBBP)	/	[321]
HACE1, SHANK2	DNA methylation	221 individuals	/	[322]
VAMP-2	Genome-wide	Mouse hippocampus	Flinders sensitive rats, MS (Maternal Separation), CUMS	[323]
245 (Set A) and 800 (Set B) significantly associated genes with antidepressant response and MDD	GWAS	SNPs from candidate genes	/	[324]
IL-6, CRP	Genome-wide methylation profiles	33 persons (blood specimens)	/	[325]
oxytocin, GABA, VGFA, TNFA, and mTOR pathways associated with suicide in the MDD group	Genome-wide methylome	Human postmortem brain samples	/	[326]
VCAN	Genome-wide association analysis	8448 individuals from UK Biobank-a population-based cohort study	/	[327]
PARK2, FGF21, HIST1H3D, RSRC1	Next-generation sequencing (NGS)	654 participants, Biological sample (saliva)	/	[328]
NEGR1, L3MBTL2, VRK2, TMEM161B, MEF2C, RERE, HACE1, LIN28B, SORCS3, OLFM4, PAX5, MEIS2, TMCO5A, RSRC1, MLF1, SLC6A15, RFX3	GWAS	45,773 cases and 106,354 controls	/	[329]
Zmynd11	GWAS	Hippocampus, striatum, frontal cortex	Carworth Farms White (CFW) mice	[330]
15 SNPs	GWAS	9240 MDD cases and 9519 controls	/	[331]
FHIT	Genome-wide association meta-analyses (GWAMA)	6,718 cases and 13,453 controls	/	[332]
147 genes significantly associated with broad depression in the total sample, 64 in the females and 53 in the males	GWAS	males (N = 127,867) and females (N = 146,274)	/	[333]
ZNF248, PRKRA, PYHIN1, SLC7A8, STK19	Exome-wide association study	182 treatment-resistant depression cases and 2021 psychiatrically healthy controls	/	[312, 334]
48 genes	Whole-genome cRNA microarray analysis	Patients, healthy controls (blood)	/	[335]
YOD1, UGT8, FNDC3B, and SLIT2 loci as new epigenetic factors associated with late-life MDD	Epigenome-wide association study (EWAS)	deceased subjects (dorsolateral prefrontal cortex)	/	[336]
MPO, FOXO1, PDE3A, TSLP, NLRP9, ADAMTS5, ROBO1, REST	GWAS	9567 participants (3510 MDD cases and 6057 controls)	/	[337]
SLC12A5, BAG5, RP11, MYRF, RP5	GWAS	807,553 European individuals (246,363 cases and 561,190 controls)	/	[338]
BDNF, IL-6, TNF-a	Methylome-wide association study (MWAS)	581 MDD patients (blood)	/	[339]
K01817, K11358, K01626, K01667	Shotgun metagenomics sequencing	26 MDD patients and 29 healthy controls (stool samples)	/	[340]
OR8B4, TRAPPC11, SBK3, TNRC6B	Exome-wide association study	120,033 participants	/	[341]
NEGR1	GWAS	16,823 MDD cases and 25,632 controls	/	[342]
PCDHA, NRG3	EWAS	One hundred sixty-six MZ twins (83 pairs)	/	[343]
STK32C	Genome-wide methylomic analysis	18 pairs of monozygotic twins, major depressive disorder (n = 14) and matched control subjects (n = 15)	/	[344]
ZNF804A, MIR3143, PSORS1C2, STK19, SPATA31D1, RTN1, TCF4	GWAS	UK Biobank	/	[345]
Cg04102384 putatively regulates gene expression of MIR4646-3p	Genome-wide methylation	depressed adults (n = 58), blood	/	[346]
rs2004237, rs34208798,	GWAS	12,310 adults, blood	/	[347]
GHSR, KCNQ1	Genome-wide DNA methylation analysis	thirty-four monozygotic twins, blood	/	[348]
DEFB1, AHR	GWAS	290 MDD patients (plasma)	/	[349]
BHLHE22	Whole-genomes	Male–male Veteran twins	/	[350]
MHC	GWAS	135,458 MDD cases and 344,901 controls (human postmortem brain dorsolateral PFC)	/	[351]
TOX2	Genome-wide analyses	21,387 subjects	/	[352]
WDR26	Genome-wide methylation study	34 participants (blood)	/	[353]
SIRT1, LHPP	GWAS	5,303 Chinese women with recurrent MDD (saliva)	/	[354]
CAPRIN1, CLEC4A, KRT23, MLC1, PLSCR1, PROK2, ZBTB16	Whole-genome microarrays	21 MDD patients, 21 healthy control subjects (blood)	/	[355]
lncRNAs located at chr10:874,695–874,794, chr10:75,873,456–75,873,642, chr3:47,048,304–47048512	Whole-genome microarrays	MDD patients (blood)	/	[356]
HOMER1	Genome-wide association studies	1738 patients and 1802 control subjects	/	[357]
317 differentially expressed genes (DEGs)	Genome-wide expression studies	Fourteen outpatients with current MDD (blood)	/	[358]
53 significantly associated novel loci	GWAS	88,316 MD cases and 902,757 controls	/	[359]
550 CpGs (MYO16)	Genome-wide analysis	Depressed (N = 19) and psychiatrically healthy controls (N = 19), prefrontal cortex	/	[360]
FGFR1, NCAM1, CAMK2A	DNA microarray analysis	Major depression (prefrontal cortex)	/	[361]
PPTC7 (cg08752433), VRS2 (cg07945879, cg14935711, cg00244776, cg15848685, cg12457901, cg16958594), VRS2 (cg26784891, cg05853013, cg04966294), MRPL46 (cg00200755), COQ8A, TRMT10C	GWAS	Major depression disorder	/	[362]
SLC25A37	GWAS	14,543 MDD cases and 14,856 controls	/	[363]

#### Applications of transcriptomics

##### Alterations in gene expression of patients with depression

Recent advancements in transcriptomics have offered a fresh perspective on depression research. Studies indicate that the gene expression profiles of individuals with depression differ significantly from those of healthy individuals. For example, the downregulation of specific genes, particularly those involved in neurotransmitter synthesis and metabolism, such as the serotonin transporter gene (SLC6A4) and BDNF, is closely linked to depression onset [364]. Alterations in the expression of these genes may disrupt neural plasticity and emotional regulation, thereby contributing to the development of depression. Additionally, the upregulation of inflammation-related genes, such as IL-6 and TNF-α, is associated with the pathological state of depression, suggesting that immune responses play a critical role in its pathogenesis [365, 366].

##### Identification of core transcription factors

In transcriptomic studies of depression, identifying key transcription factors is essential for understanding the molecular mechanisms underlying the disease. Transcription factors regulate gene expression, influencing both the physiological functions and pathological states of cells. Research has shown that factors like cAMP response element-binding protein (CREB) and nuclear factor κB (NF-κB) play pivotal roles in the pathogenesis of depression. CREB activation is linked to neural plasticity and the therapeutic effects of antidepressants, whereas NF-κB activation is associated with inflammatory responses and may exacerbate depressive symptoms [367, 368]. Additionally, transcriptomic analyses have highlighted the involvement of other transcription factors, such as STAT3 and AP-1, in depression. Changes in the expression and activity of these factors could serve as biomarkers for depression and offer potential therapeutic targets for clinical intervention [369–372].

##### Progress in spatial transcriptomics

Recent advances in spatial transcriptomics have provided new insights into the molecular mechanisms of depression, allowing researchers to identify gene modules associated with depression-like behaviors at both the cellular and cortical levels. For instance, gene expression changes related to depression predominantly occur in microglia, which play a crucial role in the development of depression-like behaviors. Specific behavioral patterns, such as social aggregation and social isolation, correlate with distinct gene expression profiles [373]. Studies also indicate that the activation patterns of particular brain regions in patients with depression, especially during emotion and reward processing, are strongly associated with gene expression patterns. These findings offer important insights into the neurobiological mechanisms underlying depression [374]. Furthermore, research has identified unique gene expression profiles in the frontal cortex of individuals with depression, which are closely linked to emotion regulation and cognitive function [375]. Spatial transcriptomic analysis of glutamatergic neurons in the brains of patients with depression has shown that their distribution in the deep medial prefrontal cortex is tightly connected to the pathogenesis of the disorder [376].

Additionally, advancements in spatial transcriptomics include high-resolution techniques, such as fluorescence in situ hybridization (FISH), in situ sequencing (ISS), and NGS, which provide cell type-specific gene expression data. These methods enable deeper exploration of the molecular foundations of depression [377]. For example, spatial transcriptomic analysis of the mouse brain revealed that depression is closely linked to gene expression changes in specific regions, including the hippocampus, isocortex, and amygdala. This research opens new avenues for the development of depression biomarkers [310]. A summary of transcriptomic research on depression is provided in Table 2.

**Table 2:** Characteristics of transcriptomics in depression

Biomarkers	Analytical technique	Samples	Model	References
NEGR1, CTC-467M3.3, TMEM106B, LRFN5, ESR2, and PROX2	Transcriptome-wide association study (TWAS)	Genome-wide association analysis of MD (n = 135,458 cases, n = 344,901 controls) and gene expression levels from 21 tissue datasets (brain; blood; thyroid, adrenal, and pituitary glands)	/	[378]
11 driver causal genes related to MDD symptoms (ESR1, RUNX1, PPARA, WWOX, etc.)	Transcriptomics	Blood samples (297 patients)	/	[315]
FOS ( Fos proto-oncogene)	Transcriptomics	79 MDD patients and 75 healthy controls; ventromedial prefrontal cortex (vmPFC) of mice	Chronic variable stress (CVS) and chronic social defeat stress (CSDS)	[379]
TP53, GR, NFκB	Genome-wide transcriptional profiling	Patients (Blood samples)	/	[380]
COL1A2, RNF150, CTGF	Transcriptome-wide	671 participants (Blood samples)	/	[381]
The differential expression of ZNF184 was strongest in subcortical regions in males and females	Transcriptomic	246,363 cases (human postmortem brain tissues)	/	[312]
Depressed mood was linked to upregulated expression of inflammation-related genes. Depressed mood was linked to downregulated expression of antiviral-related genes	Transcriptomic	Participants (n = 87), blood samples	/	[382]
RGS2	Transcriptome	California mice (Peromyscus californicus), nucleus accumbens (NAc)	Social Defeat Stress (SDS)	[383]
ExN4_L35, ExN7, ExN12_L56, ExN13_L56, InN10_ADARB2	Single-nucleus RNA-sequencing	71 female and male donors (dorsolateral prefrontal cortex)	/	[384]
six enriched gene modules	Single-nucleus RNA-sequencing, spatial transcriptomics	Female cynomolgus macaques (dorsolateral prefrontal cortex)	Social stress-associated depressive-like behaviors	[373]
26 cellular clusters	Single-nucleus transcriptomics	Male individuals with MDD (n = 17) and of healthy controls (n = 17), dorsolateral prefrontal cortex	/	[385]
HBEGF, CXCL8	Transcriptomic profiles	Female patients (Blood)	/	[386]
Malat1	Single-nucleus transcriptomic analysis	17 healthy controls (HC) and 17 MDD cases, dorsolateral prefrontal cortex	/	[387]
MTRNRL8, IL8, SERPINH1, CCL4	NGS whole-transcriptome profiling (RNA-seq)	Patients (dorsolateral prefrontal cortex)	/	[388]
GAL, NPY, TAC1, CCK, TACR1, Cerebellin4, CARTPT, Adcyap1R1, GPR173	Single-nucleus RNA-sequencing	Patients (dorsolateral prefrontal cortex, anterior cingulate cortex, dorsal raphe nucleus, locus coeruleus, medullary raphe nucleus)	/	[389]
104 genes	RNA-sequencing (RNA-seq)	Patients (dorsolateral prefrontal cortex)	/	[390]
NECAB2	RNA-sequencing	Patients (dorsomedial prefrontal cortex)	/	[391]
TRPV2, ZNF713, CTSL	RNA-sequencing	Patients (peripheral blood mononuclear cells)	/	[392]

#### Role and value of metabolomics

##### Metabolic characteristics of patients with depression

Metabolomics plays a crucial role in depression research by revealing the relationship between the metabolic characteristics of patients and the disease. Studies indicate that the metabolic profiles of individuals with depression often differ significantly from those of healthy controls. For instance, depression affects energy metabolism, leading to abnormalities in lipid and amino acid metabolism. These metabolic alterations may be closely linked to the pathophysiological mechanisms underlying depression [393, 394]. In one study, mass spectrometry was used to analyze body fluid samples from patients with depression, revealing significant changes in various metabolites, including long-chain fatty acids and amino acids. These alterations may correlate with the severity of depression and the response to treatment [395–397]. Moreover, metabolomics has identified distinctive changes in inflammatory markers and energy metabolism-related metabolites in individuals with depression, further supporting the potential of metabolomics as a valuable tool in depression research [398–401]. By monitoring these metabolites, clinicians can gain a deeper understanding of the changes in patients' conditions and tailor treatment plans accordingly [14].

##### Integrated analysis of metabolomics and other omics

The integration of metabolomics with other omics, such as genomics, transcriptomics, and proteomics, offers a new approach to depression research. For example, the integration of metabolomics and transcriptomics has revealed a strong correlation between specific metabolic pathways and changes in gene expression, providing valuable insights into the biological mechanisms of depression [402]. Moreover, this integrated approach can help identify key biomarkers associated with depression, facilitating the development of precision medicine [403]. By combining data across various omics layers, researchers can capture the multidimensional aspects of depression, offering a solid foundation for the design of personalized treatment strategies [14, 404]. This multi-omics integration not only deepens our understanding of depression but also paves the way for future therapeutic advancements.

##### Spatial distribution of metabolites in the brain

Spatial metabolomics, an emerging technology, enables the examination of the spatial distribution of metabolites at the tissue level, offering crucial insights into the mechanisms underlying depression. For example, researchers have used mass spectrometry imaging to map metabolite distribution in brain tissue from mice. This approach revealed concentration changes in specific metabolites, such as amino acids and lipids, in the brains of depression model mice, suggesting a link to the pathophysiological changes associated with depression [405].

The application of spatial metabolomics allows for detailed analysis of metabolic alterations in specific brain regions affected by depression. For instance, a study employing mass spectrometry imaging found significant differences in metabolite distribution in the cerebellar cortex, prefrontal cortex, and hippocampus of depression model mice. These regional variations may correlate with cognitive and emotional dysfunctions observed in depression [406]. Such region-specific metabolic changes offer a novel perspective on the neurobiology of depression and could serve as potential targets for future therapeutic interventions. A summary of metabolomics research on depression is provided in Table 3.

**Table 3:** Characteristics of metabolomics in depression

Biomarkers	Analytical technique	Samples	Model	References
98 metabolites	GC–MS	Left hippocampus of rats	CUMS	[407]
50 fecal metabolites	GC–MS	Subjects feces	/	[408]
thirty-seven differentially expressed metabolites	LC–MS	SD rats (brains)	CSDS	[409]
Twelve metabolites were significantly increased (glycine and adenosine) or decreased (3-hydroxybutyric acid, creatinine, 2,5-dihydroxypyrazine, pantothenic acid, dihydroxyacetone phosphate, proline, phenylalanine, tyrosine, lysine, and glutamine) in depressed rat cerebella	GC–MS	SD rats (cerebella)	Chronic mild stressed (CMS)	[410]
42 metabolites	LC–MS/MS	Rats (amygdala)	CUMS	[411]
191 differential metabolites	NMR, GC–MS, LC–MS	Fecal microbiota transplantation mouse livers	CUMS	[412]
31 metabolites	Quantitative, targeted liquid chromatography	290 MDD patients (plasma)	/	[349]
30 metabolites	GC–MS	Rat (hippocampus)	CUMS	[413]
Twelve metabolites	GC–MS	Rat (plasma)	CUMS	[414]
49 metabolites	^1^H-NMR	6811 individuals with lifetime MDD and 4370 with recurrent MDD (blood)	/	[415]
Five amino acid metabolic (tyrosine, glycerophospholipid metabolism)	HILIC-MS, RPLC-MS	53 adolescents diagnosed with first-episode drug-naïve MDD (FEDN-MDD), 53 adolescents with TRD, and 56 healthy controls, plasma	/	[416]
Eight metabolites	^1^H-NMR	Rat (plasma)	CUMS	[417]
14 metabolites (ornithine, beta-alanine)	LC-TOFMS	Participants (urinary)	/	[418]
several fatty acids, glycerol and gamma-aminobutyric acid (GABA)	GC–MS	Nine depressed, 11 remitted, and ten never-depressed older adults (plasma)	/	[419]
79 metabolites	UPLC-MS/MS	2770 subjects, 1805 subjects (plasma)	/	[420]
D-pinitol, indoxyl sulfate, trimethylaminen-oxide, 3 alpha,7 alpha-dihydroxy-12-oxocholanoic acid	UPLC-MS/MS	Rat (stool)	CUMS	[421]
Glutamate	LC–MS/MS	Mice (prefontal cortex)	CSDS	[422]
Four fatty acid	UPLC-QTOF-MS	57 unmedicated patients with major depressive disorder (MDD) and 57 healthy controls, serum and urinary	/	[423]
Leucine	/	Mice	CSDS	[424]
Two hundred eight metabolites	UHPLC-QTOF-MS	65 patients and 65 healthy controls (serum), rat (serum, CSF, hippocampus)	CUMS	[425]
19 metabolites	GC–MS	rat (olfactory bulb)	CUMS	[426]
l-Kynurenine	LC–MS/MS	MDD (n = 49) and HC (n = 68), plasma	/	[427]
Spermine, leucine, propionylcarnitine, butyrylcarnitine, hippuric acid, methyl-hippuric acid, cholic acid, CDCA, LPC C16:0	UFLC/MS-IT-TOF	Rat (plasma, urine)	Chronic restraint stress (CRS)	[428]
hypoxanthine, phytosphingosine, xanthine	LC/GC‒MS	379 individuals, (plasma)	/	[429]
Amino acids, fatty acids, bile acids, hypoxanthine, stercobilins	UHPLC-MS	Rat (fecal)	CVS	[430]
N-acetylaspartate	^1^H-NMR	Rat (hippocampus)	CMS	[431]
L-glutamine, L-tyrosine, hydroxylamine, 3-phosphoglyceric acid	GC–MS	Rat (hippocampus, jejunum)	CUMS	[432]
44 differential metabolites	UPLC‐QTOF‐MS	Mouse (Hippocampal)	Lipopolysaccharide-induced mouse model	[433]
TMA, aspartic acid, glutamate, AcAc, NAc, alanine, lactate, Leu/Ile, lipids, proline, β-HB, valine	^1^H-NMR	Rat (plasma)	Acute and chronic stress models	[434]
468 differential metabolites	LC–MS	Rat (fecal)	CUMS, CRS, social defeat, learned helplessness	[435]
Fifteen metabolites	^1^H-NMR, UPLC‐QTOF‐MS	Rat (urinary)	CUMS	[436]
20 differential metabolites	GC–MS	Mouse (prefrontal cortex)	Lipopolysaccharide-induced mouse model of depression	[437]
10 differential metabolites	NMR, GC–MS	Young and middle-aged MDD patients (urine)	/	[438]
18 differential metabolites	GC–MS	Rat (Peripheral blood mononuclear cells)	CUMS	[439]
Glutamate, ornithine aspartic acid, 5-hydroxytryptophan, L-tyrosine, norepinephrine, homovanillic acid	LC–MS	Mouse (entorhinal cortex)	CRS	[440]

GC–MS: gas chromotography/mass spectrometry

LC–MS: liquid chromatography mass spectrometry

LC–MS/MS: liquid chromatography-tandem mass spectrometry

NMR: nuclear magnetic resonance

HILIC-MS: liquid chromatography-mass spectrometry

RPLC-MS: reversed-phase liquid chromatography-mass spectrometry

LC-TOFMS: LC-time-of-flight mass spectrometry

UPLC-MS/MS: ultra performance liquid chromatography tandem mass spectrometry

UPLC-QTOF-MS: ultra-performance liquid chromatography quadrupole/time of flight-mass spectrometry

UHPLC-QTOF-MS: ultra-high performance liquid chromatography quadrupole/time of flight-mass spectrometry

UFLC/MS-IT-TOF: fast liquid chromatography coupled with ion trap-time of flight mass spectrometry

UHPLC-MS: ultra high-performance liquid chromatography-mass spectrometry

#### New perspectives provided by proteomics

##### Discovery of protein markers

In depression research, the application of proteomics allows for the identification of potential biomarkers, which are crucial for the early diagnosis and personalized treatment of the disorder. For example, studies have shown that neuron-specific proteins, such as nerve growth factor and BDNF, exhibit significant changes in expression in both the blood and brain tissues of individuals with depression. These alterations may reflect underlying pathophysiological mechanisms of the disease [393, 441, 442]. Additionally, high-throughput proteomics has revealed proteins involved in immune responses and inflammation, including interleukin-6 (IL-6) and tumor necrosis factor-α (TNF-α). The levels of these proteins are notably elevated in patients with depression, suggesting their critical role in the disorder's pathogenesis [443–445].

##### Association between proteomics data and clinical characteristics

Proteomics analyses not only identify biomarkers associated with depression but also uncover critical links between these biomarkers and clinical characteristics. By integrating proteomic profiles from patients with depression, researchers have found that the expression levels of specific proteins significantly correlate with clinical symptoms, including depression severity and anxiety levels. For example, several studies report that elevated levels of blood-based markers such as fibrinogen are positively associated with the intensity of depressive symptoms. These insights offer promising biomarkers for evaluating both the severity of depression and patient responses to treatment [446, 447]. In addition, proteomic studies highlight the complex interplay between depression and comorbid conditions such as diabetes, emphasizing the importance of accounting for a patient’s overall health status during diagnosis and treatment planning [448].

##### Construction of protein interaction networks

The construction of protein interaction networks offers a powerful framework for exploring the molecular mechanisms underlying depression. By integrating proteomic and genomic data, researchers can map interaction networks among depression-associated proteins. These networks not only clarify the roles of key proteins in the pathogenesis of depression but also help identify novel therapeutic targets. For example, studies have shown that several signaling pathways implicated in depression—such as the PI3K-Akt and MAPK pathways—occupy central positions within depression-related protein–protein interaction networks. Core components of these pathways, including AKT1 and TNF, have emerged as potential targets for therapeutic intervention [449]. Moreover, the application of machine learning and network analysis enables the identification of hub proteins from complex interaction maps, offering a data-driven foundation for personalized treatment strategies [450].

##### Rise of spatial proteomics

In the context of depression, spatial proteomics allows for the investigation of region-specific protein distribution patterns, providing insights into how molecular changes vary across neural circuits. This approach offers a new dimension for studying depression, deepening our understanding of its underlying mechanisms and revealing new molecular targets. Ultimately, spatial proteomics lays the groundwork for future clinical applications by informing both diagnostics and treatment strategies. A summary of key proteomic findings in depression is presented in Table 4.

**Table 4:** Characteristics of proteomics in depression

Biomarkers	Analytical technique	Samples	Model	References
529 proteins	iTRAQ	Right hippocampus of rats		[407]
Identified 159, 187, 148, and 55 DE proteins in the prefrontal cortex (PFC), liver, cecum, and serum, respectively	iTRAQ	Mice (prefrontal cortex, liver, cecum, and serum)	Humanized fecal microbiota transplantation (FMT)	[451]
274 DE and 201 DE proteins	LC–MS/MS	Mouse hippocampus	CUMS	[317]
BTN3A3, PSMB4, TIMP4, ITIH1	pQTL	Human plasma	/	[318]
NCAM1, NRCAM, NTRK3	SOMAscan	52 women (plasma)	/	[452]
Insulin, MMP-9	Proteomic approach	245 depressed patients (plasma)	/	[453]
123 proteins	iTRAQ	SD rats (brains)	CSDS	[409]
AACT, APOE, APOH, FETUA, HBA, PHLD	LC–MS/MS	MDD patients (Blood serum samples)	/	[454]
1815 proteins	iTRAQ	SD rats (cerebella)	CMS	[410]
IL-6, STAT3, PRDM1, PPARGC1A	Quantitative mass spectrometry	15 patients (cerebrospinal fluid samples)	/	[444]
C-reactive protein, leptin, insulin	Proteomic analytes	1621 subjects (Serum)	/	[455]
Lrrc8d, Dcun1d2, Mtnd5, Ccdc154, Sec14l2, Kif2a, LOC680322, Me1, Mknk1, Ret7, Sec14l2, Serpinf2, LOC103694855, Fam13c, Loxl1	TMT-labelled quantitative proteomics	Medial prefrontal cortex (mPFC), nucleus accumbens (NAc), and hippocampus (Hip)	Wistar Kyoto (WKY) rats	[456]
C3, CFI, C4BPα, kininogen-1, antithrombin	MALDI-TOF/TOF MS	Bipolar disorder (BD) and MDD patients (plasma samples)	/	[457]
96 differentially expressed proteins	Untargeted mass spectrometry	19 with late-life depression (LLD) and 31 controls, plasma samples	/	[458]
Fibrinogen, kininogen	MALDI-TOF MS	86 patients and 89 healthy controls, serum samples	/	[446]
pancreatic polypeptide, prostasin, luteinizing hormone, AAT, macrophage migration inhibitory factor (MIF), GROa, fetuin-A	Proteomic analytes	1589 participants, serum	/	[459]
Twenty-nine differential proteins	Matrix-assisted laser desorption ionization-time of flight-tandem mass spectrometry	CUMS rat, prefrontal cortex (PFC)	CUMS	[460]
902 proteins	i-TRAQ	Monkey models and human MDD patients (cerebrospinal fluid)	CMS	[461]
Thirty-three proteins were uniquely associated with LLD in females, while six proteins were uniquely associated with LLD in males	Untargeted proteomic analysis	430 individuals with LLD and 140 healthy comparisons (plasma)	/	[462]
171 proteins	iTRAQ	Rats (amygdala)	CUMS	[411]
Thirty-seven proteins	MALDI-TOF–MS/MS	Mice (Hypothalamus)	CUMS	[463]
CRP, ATIII, ITIH4, VDB	proteomics study	7 MDD patients and 7 healthy controls (plasma)	/	[464]
NRXN3, CNTNAP4, GRIA4	iTRAQ	MDD patients (n = 40) and controls (n = 27), CSF	/	[465]
Twenty nine differential protein	MALDI-TOF MS/MS	Rat (olfactory bulb)	CUMS	[466]
23, 29, and 30 proteins	LC–MS	675 subjects (plasma)	/	[467]
P2RX7, Caspase 1, ASC, IL-1β	iTRAQ	Mice (PFC)	CSDS	[468]
Nine proteins	iTRAQ, LC-chip-MS/MS	Depressed patients and healthy controls (plasma)	/	[469]
PRDX2	iTRAQ, nano-LC–MS/MS	Patients (plasma)	/	[470]
ROS, ER	MALDI-TOF MS	Mice (whole brain)	CUMS	[471]
F13A1, PPBP, PF4, GAPDH, TMSB4X	LC–MS	Patients (plasma)	/	[472]
367 proteins	iTRAQ, LC–MS/MS	Mice (olfactory bulb)	CUMS	[473]
Bacteroidetes, Proteobacteria, Firmicutes, Actinobacteria	iTRAQ, tandem mass spectrometry	MDD patients (feces)	/	[474]
Nine proteins	MRM-MS	270 individuals (plasma)	/	[475]
170 proteins	iTRAQ	Rat (hippocampus)	CUMS	[413]
73 proteins (SCN9A)	iTRAQ, LC–MS	Rat (hippocampus)	CUMS	[476]
ApoA-IV, ApoC-II, CRP, LRG	iTRAQ, LC–MS/MS	Patients, healthy control subjects (plasma)	/	[477]
mesothelin, leptin, IGFBP1, IGFBP2, FABPa, insulin, C3, B2M	Proteomic profiles	414 controls, 231 persons with a melancholic depressive subtype and 128 persons with an atypical depressive subtype (serum)	/	[478]
HINT1	Shotgun proteomics, Nano-high-performance liquid chromatography-mass spectrometry analyses	24 MDD patients and 12 matched controls (post-mortem brains)	/	[479]
CRP, ITIH4, SAA1, ANGPTL3	iTRAQ, LC–MS/MS	22 depressed patients and 20 healthy controls (serum)	/	[480]
OGDH, SDHA, COX5B, PFN1, HSP90AA1, PDCD6IP, DBN1, DBNL, MYH9	LC–MS/MS	BD and of MDD patients, dorsolateral prefrontal cortex (DLPFC) and anterior cingulate cortex (ACC)	/	[481]
47 differentially expressed proteins	iTRAQ, LC–MS	Mouse (plasma)	CUMS	[482]
26 differentially expressed proteins (creatine kinase B, dihydropyrimidinase-like 3)	2-DE, MALDI-TOF–MS/MS	Mouse (prefrontal cortex)	Lipopolysaccharide-induced mouse model	[483]
74 proteins	iTRAQ, LC–MS/MS	Rat (hippocampi)	CMS	[484]
74 differential proteins	iTRAQ, MS	Rat (hippocampus)	CUMS	[485]
98 differential proteins	iTRAQ, HPLC–MS	Rat (Liver)	CRS	[486]
81 differential proteins	2‐DE	Mouse (Hippocampal)	Lipopolysaccharide-induced mouse model	[433]

iTRAQ: isobaric tags for relative and absolute quantitation

LC–MS/MS: liquid chromatography-tandem mass spectrometry

MALDI-TOF/TOF MS: matrix-assisted laser desorption/ionization-time-of-flight/time-of-flight tandem mass spectrometry

MALDI-TOF MS: matrix-assisted laser desorption ionization time of flight mass spectrometry

MALDI-TOF–MS/MS: matrix-assisted laser desorption ionization-time of flight-tandem mass spectrometry

MRM-MS: targeted proteomic approach, Multiple reaction monitoring-mass spectrometry

LC–MS: liquid chromatography mass spectrometry

2-DE: proteomes by two-dimensional electrophoresis

HPLC–MS: high performance liquid chromatography and mass spectrum

#### Association between microbiomics and depression

##### Correlation between microbiomics and depressive symptoms

In recent years, the relationship between the microbiome composition and depressive symptoms has become a major area of research. Studies have demonstrated that gut microbiota influence host mental health through multiple mechanisms, including neurotransmitter production, immune system modulation, and regulation of intestinal barrier function [487, 488]. For example, certain intestinal bacteria can synthesize 5-hydroxytryptamine (5-HT), and its levels are closely linked to the severity of depressive symptoms [489, 490].

Numerous clinical studies comparing the gut microbiomes of patients with depression to those of healthy controls have revealed significant differences in microbiome composition. Specifically, these studies show a reduction in the diversity of gut microbiota in patients with depression, along with altered abundances of specific bacterial communities. These microbial changes may contribute to the pathogenesis of depression [489, 491]. Further research has indicated that shifts in the abundance of specific bacteria, such as Firmicutes and Eggerthia, correlate with the severity of depressive symptoms, suggesting that microbial changes can influence both mood and cognitive function [490, 492]. Additionally, certain microbial communities, such as the Prevotella microbiome, appear to be associated with milder depressive symptoms, implying that specific microbiota may offer a protective effect against depression [488]. In a systematic review, researchers analyzed the gut microbiomes of patients with depression and found substantial differences in microbiome composition compared to healthy individuals, particularly in the abundance of certain bacterial genera, such as Prevotellaceae and Blautia [493, 494].

##### Microbiomes affect neurotransmitters and inflammatory responses in depression

Studies show that gut microbiota produce a variety of bioactive metabolites that can influence neurotransmitter levels in the host, thereby playing a crucial role in regulating emotions and behaviors. For example, gut microbiota can affect the synthesis and release of neurotransmitters by producing short-chain fatty acids (SCFAs) such as acetic acid, propionic acid, and butyric acid. These SCFAs not only serve as an energy source but also regulate nervous system functions by binding to host cell receptors, influencing both emotional and cognitive processes [491].

Changes in microbiome composition are also closely associated with depression. Research has demonstrated that gut microbiota diversity is significantly reduced in individuals with depression, and the abundance of specific bacterial families, such as Lachnospiraceae and Veillonellaceae, negatively correlates with the severity of depressive symptoms [490]. These microorganisms may influence emotional states by modulating tryptophan metabolism and altering the synthesis of neurotransmitters like 5-hydroxytryptamine (5-HT) [284, 495–497]. Specifically, tryptophan, a precursor of 5-HT, is metabolized by intestinal microbiota. An imbalance in the microbiota may shift the metabolic pathway of tryptophan, leading to a reduction in 5-HT production and contributing to the onset of depressive symptoms [498–500].

In addition, the microbiome indirectly regulates neurotransmitter balance by modulating the host's immune response and inflammatory state. Depression is frequently associated with chronic low-grade inflammation, and gut microbiota influence the intensity of this inflammatory response by affecting immune cell activity and cytokine secretion. For example, the genus Prevotella is often found in lower abundance in individuals with depression and is linked to elevated levels of inflammatory markers such as TNF-α [501]. These findings suggest that the microbiome may contribute to the pathogenesis of depression through complex interactions involving microbial metabolites, neurotransmitter regulation, and immune system modulation.

##### Application of microbiome intervention in depression

*Clinical trials of probiotics and depression*. In recent years, probiotics have gained attention as an important microbiome intervention, particularly in the treatment of depression. Clinical trials demonstrate that probiotics can alleviate depressive symptoms by modulating the gut microbiome. Research has shown that patients with depression often exhibit significant microbiome imbalances, characterized by a reduction in beneficial bacteria and an increase in harmful ones [489, 491]. Supplementing with probiotics may help restore gut microbiota balance, thereby improving mental health.

In a randomized controlled trial, researchers implemented a 31-day probiotic intervention for patients with depression. The results indicated a significant reduction in depressive symptoms, along with an increase in gut microbiota diversity, particularly a notable enhancement in the abundance of lactic acid bacteria [502]. Additionally, another study found that probiotic supplementation not only improved depressive symptoms but also modulated neural mechanisms associated with emotional responses by influencing functional brain connectivity [503].

While current evidence suggests that probiotics may have a beneficial impact on depression, further high-quality clinical trials are needed to confirm their efficacy. Additionally, more research is required to understand the mechanisms of action of different probiotic strains and determine their optimal dosing regimens [504, 505].

*Impact of diet on the microbiome*. Diet plays a critical role in shaping the composition and function of the gut microbiome. Recent studies have shown that dietary intake can directly influence microbial diversity and abundance, while also indirectly affecting mental health by altering microbial metabolite profiles. For example, diets rich in fiber promote the growth of beneficial bacteria, thereby increasing the production of SCFAs, which have been shown to exert antidepressant effects [506, 507].

In patients with depression, researchers have observed a positive correlation between dietary patterns and gut microbiota diversity. Individuals adhering to healthy dietary habits tend to exhibit greater microbial diversity and experience milder depressive symptoms [487, 508, 509]. Notably, the Mediterranean diet is widely regarded as one of the most beneficial patterns for promoting gut microbiome health and has been shown to significantly reduce the risk of depression [510–512].

Moreover, dietary changes can exert rapid and substantial effects on the microbiome. Even short-term modifications in diet can lead to significant shifts in microbial composition, offering promising strategies for improving mental health through nutritional intervention [513]. Therefore, future studies should prioritize investigating the dynamic interactions between diet and the microbiome to develop more effective, microbiome-targeted treatments for depression.

##### Inspiration from spatial microbiomics

The integrated analysis of spatial microbiome offers a novel perspective for understanding the complex relationship between the microbiome and depression. For instance, high-throughput sequencing technologies enable detailed comparisons of microbial composition between patients with depression and healthy controls. Spatial analysis techniques further allow investigators to examine the distribution of these microorganisms across different intestinal regions and assess their interactions with host metabolic pathways. Moreover, spatial microbiome analysis can identify location-specific associations between microbial communities and depressive symptoms, offering a foundation for precision medicine and personalized treatment strategies. This integrative approach not only advances our understanding of the pathophysiological mechanisms underlying depression but also supports the development of targeted clinical interventions. A summary of microbiome research related to depression is provided in Table 5.

**Table 5:** Characteristics of microbiomics in depression

Biomarkers	Analytical technique	Samples	Model	References
Tryptophan and glutamate synthesis modules and the 3,4-Dihydroxyphenylacetic acid synthesis module (related to dopamine metabolism) were -negatively associated with MDD and/or dysthymia	Metagenomes	Participants stool	/	[514]
3 bacteriophages, 47 bacterial species	Metagenomes	Participants stool	/	[408]
Bacteroides, Clostridium, Bifidobacterium, Oscillibacter, Streptococcus	Shotgun metagenomics sequencing	Thirty one MDD patients, thirty BPD patients, and thirty healthy controls (faecal)	/	[515]
Faecalibacterium prausnitzii	shotgun metagenomic study	133 individuals with depression and 532 without depression (stool)	/	[516]
The phylum Bacteroidetes abundance was significantly reduced, whereas that of the Actinobacteria and Firmicutes were significantly increased in patients	Shotgun metagenomics sequencing (SMS)	25 patients and 28 healthy controls (faecal)	/	[517]
Phyla Proteobacteria/Pseudomonadota and Bacteroidetes/Bacteroidota	16S RNA sequencing	6811 individuals with lifetime MDD and 4370 with recurrent MDD (blood)	/	[415]
Decreased the ratio of Firmicutes to Bacteroidetes	16S RNA sequencing	Rat (faecal)	CUMS	[417]
Alistipes indistinctus, Bacteroides ovatus, Alistipes senegalensis	16S RNA sequencing	Rat (stool)	CUMS	[421]
Firmicutes, Bacteroidetes, Proteobacteria, Actinobacteria, Verrucomicrobia, Fusobacteria	16S RNA sequencing	57 unmedicated patients with major depressive disorder (MDD) and 57 healthy controls, fecal	/	[423]
Marvinbryantia, Corynebacterium, Psychrobacter, Christensenella, Lactobacillus, Peptostreptococcaceae incertae sedis, Anaerovorax, Clostridiales incertae sedis, Coprococcus, Candidatus Arthromitus, Oscillibacter	16S RNA sequencing	Rat (fecal)	CVS	[430]
Bacteroidota, Firmicutes, Cyanobacteria, Patescibacteria	16S RNA sequencing	Mice (fecal)	CRS	[518]
Lachnospiraceae, Muribaculaceae, Oscillospiraceae	16S RNA sequencing	Rat (fecal)	CUMS, CRS, social defeat, learned helplessness	[435]
Bacteroidetes, Proteobacteria, Actinobacteria, Firmicutes	High-throughput pyrosequencing	46 patients (fecal)	/	[519]
phylum Firmicutes	16S RNA sequencing	Mice (fecal)	CSDS	[520]
Firmicutes, Bacteroidetes, Actinobacteria	16S RNA sequencing	70 MDD patients and 71 healthy controls (HCs), fecal	/	[521]
Escherichia-Shigella, Flavonifractor	16S RNA sequencing	101 depressive patients and 70 controls (fecal)	/	[522]
Lactobacillus, Bifidobacterium, Romboutsia, Staphylococcus, Psychrobacter, Corynebacterium	16S RNA sequencing	Mice (fecal)	CUMS	[523]

### Application of omics in the discovery of antidepressant chinese medicine targets

#### Genomic identification of antidepressant chinese medicine targets

##### Screening methods for targets

The screening methods for identifying targets of antidepressant Chinese medicine include gene expression analysis, network pharmacology, and protein interaction network analysis. Gene expression analysis compares the differential expression of genes between patients with depression and healthy controls, enabling the identification of key genes associated with depression. For example, researchers identified 3,533 depression-related genes through analysis of the GEO database, which provided a foundation for subsequent target screening in Chinese medicine [246]. Network pharmacology offers a comprehensive approach to understanding the multi-target actions of Chinese medicine. By constructing a network linking drugs, targets, and diseases, researchers can uncover how Chinese medicine components exert antidepressant effects. Using this approach, several potential targets have been identified, including PRKACA, NCOA2, and PPARA, which play crucial roles in regulating neuroendocrine function, metabolism, and neuro-immunity [246].

Additionally, protein interaction network analysis is widely used for target screening. By examining the interactions among various targets, researchers have identified key hub genes—such as ACHE, IL6, SLC6A4, and FOS—that are critical in the pathogenesis of depression treated with Chinese medicine [524]. Combining these methods provides a more comprehensive understanding of the mechanisms through which Chinese medicine combats depression, thereby offering a scientific basis for selecting appropriate therapeutic targets.

##### Advantages of genomics in target research

Genomics offers significant advantages in identifying targets for antidepressant Chinese medicine, particularly through high-throughput screening, precise target localization, and multi-dimensional analysis. First, genomics enables high-throughput screening of numerous genes, which is essential for identifying potential antidepressant targets. For example, RNA sequencing technology allows researchers to analyze tens of thousands of gene expression changes simultaneously, facilitating the rapid identification of key genes related to the efficacy of Chinese medicine in treating depression [525, 526]. Second, genomics provides precise target localization. Through GWAS, researchers can pinpoint specific genetic variations associated with depression. These variations may influence neurotransmitter synthesis and metabolism, ultimately affecting mood and behavior [527, 528]. This precise targeting capability allows researchers to more effectively select candidates for further functional validation and drug development.

Finally, genomics enables multi-dimensional analysis by integrating transcriptomic, proteomic, and metabolomic data. This comprehensive approach reveals the complex mechanisms through which antidepressant Chinese medicine exerts its effects. Studies have shown that Chinese medicine affects multiple pathways, including the regulation of nerve growth factors, inflammatory responses, and endocrine axes [232, 529, 530]. The ability to uncover these multi-target and multi-pathway mechanisms is a unique strength of genomics technology. Therefore, the application of genomics offers new insights and methodologies for researching antidepressant targets in Chinese medicine, advancing the modernization and precision of Chinese medicinal therapies. A summary of genomics applications in antidepressant Chinese medicine is provided in Table 6.

**Table 6:** Characteristics of the application of genomics in antidepressant Chinese medicine

Drug	Biomarkers	Analytical technique	Samples	Model	References
Xiaoyao Pills	DNMT1	Genome-wide DNA methylation sequencing	Patients and healthy (blood)	/	[526]
Xiaoyaosan	Lmx1b, Abcc5, Gpc3, Cfb	Whole-genome bisulfite sequencing (WGBS)	Rat (arcuate nucleus of hypothalamu)	CUMS	[531]

#### Transcriptomic characteristics of antidepressant chinese medicine targets

##### Identification of antidepressant targets

Transcriptomic analysis enables researchers to identify key depression-related genes by examining gene expression changes in animal or human depression models. For example, studies have shown that the active compounds in the Chinese medicine Yueju exert antidepressant effects by upregulating the expression of pituitary adenylate cyclase-activating polypeptide (PACAP) in the hippocampus [532]. The therapeutic mechanisms of antidepressant Chinese medicines involve various genes and signaling pathways. Research indicates that neuroinflammation, BDNF signaling, and monoamine neurotransmitter metabolism are closely associated with the pathophysiology of depression. Specific components of Chinese medicine can enhance neuronal growth and survival by modulating BDNF and its downstream signaling cascades, thereby alleviating depressive symptoms [533–535].

Additionally, transcriptomic studies have uncovered multiple signaling pathways involved in the antidepressant actions of Chinese medicine. These pathways serve critical regulatory functions. For instance, certain herbal compounds alleviate depression by activating the Creb1/Six3os1 axis or modulating the Ghrl–Edn1/Mecp2/P-mTOR/VEGFA pathway [536, 537]. By systematically analyzing these key genes and their regulatory networks, researchers can gain a deeper understanding of the molecular mechanisms underlying traditional antidepressant therapies and establish a scientific foundation for new drug development.

##### Animal experiments and clinical verification

Animal experiments serve as a cornerstone in the study of antidepressant Chinese medicines. By establishing validated depression models, researchers can systematically assess both therapeutic efficacy and underlying mechanisms. For example, using the chronic unpredictable mild stress (CUMS) model, studies have demonstrated that specific Chinese medicines significantly improve depressive-like behaviors in animals. Transcriptomic analysis has further verified their molecular targets and mechanisms of action [538].

Clinical validation holds equal importance. Numerous clinical trials have confirmed the antidepressant effects of Chinese medicine formulations. For instance, studies involving patients with depression have shown that certain compound prescriptions effectively alleviate depressive symptoms while maintaining favorable safety profiles [525, 526]. By integrating findings from animal models and clinical research, we can more comprehensively evaluate the efficacy of Chinese medicines, ultimately facilitating their clinical translation. Spatial transcriptomics offers an advanced tool for identifying drug targets. This approach enhances the precision of target identification and helps map the complex interaction networks of bioactive components. Notably, some studies have used spatial transcriptomics to reveal that antidepressant Chinese medicines exert their effects by modulating genes involved in neuroinflammation and neuroplasticity [539]. The application of transcriptomics, animal models, and spatial analysis techniques has introduced novel strategies for elucidating their mechanisms of action. A summary of the application of transcriptomics in antidepressant Chinese medicine is presented in Table 7.

**Table 7:** Characteristics of the application of transcriptomics in antidepressant Chinese medicine

Drug	Biomarkers	Analytical technique	Samples	Model	References
Total ginsenoside ginseng root (TGGR)	32 DEGs	RNA sequencing	Mice (hippocampal)	CUMS	[540]
20(S)-protopanaxatriol (PPT)	350 DEGs	RNA sequencing	Mice (hippocampal)	CUMS	[541]
Xiaoyaosan	Lmx1b, Abcc5, Gpc3, Cfb	RNA sequencing	Rat (arcuate nucleus of hypothalamu)	CUMS	[531]

#### Metabolomics identification of antidepressant chinese medicine targets

##### Metabolomics strategies for identifying targets

Metabolomics plays a crucial role in identifying the molecular targets of Chinese medicines used to treat depression. Studies utilizing techniques such as liquid chromatography–mass spectrometry (LC–MS) have revealed significant metabolic alterations in animal models of depression. For instance, Chaigui granules demonstrated notable antidepressant effects in a CUMS model. Metabolomic profiling revealed that the granules modulated several key metabolic pathways, including purine metabolism, thereby offering important leads for target identification [542]. Furthermore, integrating metabolomics with network pharmacology enhances our understanding of the multi-target nature of Chinese medicine formulations. This combined approach elucidates the complex interactions between bioactive compounds and biological networks, ultimately helping to uncover the mechanisms underlying their antidepressant effects [543].

##### Metabolite characteristics analysis of related targets

In metabolomics research, identifying the targets and their associated metabolites linked to antidepressant Chinese medicine is essential. Studies have demonstrated that Radix Bupleuri and Radix Paeoniae Alba influence depression through various metabolic pathways, including amino acid and energy metabolism [544–546]. Metabolomics analysis has also shown that Chinese medicine can significantly alleviate depressive symptoms induced by CUMS and modulate biomarkers such as BDNF and the HPA axis [546]. This characterization of metabolite profiles not only deepens our understanding of the mechanisms behind Chinese medicine’s effects but also provides a theoretical foundation for its clinical use.

##### Target verification in clinical application

Verifying the efficacy and safety of Chinese medicine targets for antidepressant effects in clinical settings is crucial. By integrating metabolomics data with clinical trial outcomes, researchers can more accurately assess the real-world impact of Chinese medicine on depression treatment. For example, Changpusan has demonstrated significant antidepressant effects in the CUMS model, with its impact on tryptophan metabolism confirmed through metabolomic profiling [547]. Furthermore, combining network pharmacology with metabolomics allows for more robust verification of the multi-target mechanisms underlying Chinese medicine’s antidepressant properties, thus strengthening the scientific foundation for its clinical application [548]. Through such studies, metabolomics not only facilitates the identification of potential targets for antidepressant Chinese medicines but also paves the way for their broader clinical adoption. A summary of metabolomics applications in antidepressant Chinese medicine is presented in Table 8.

**Table 8:** Characteristics of the application of metabolomics in antidepressant Chinese medicine

Drug	Biomarkers	Analytical technique	Samples	Model	References
Jiaotai Pills	32 metabolites	LC–MS	Mice (serum)	CUMS	[549]
Xiaoyaosan	Nine metabolites	^1^H NMR	Rat (kidney)	CUMS	[550]
Cistanche deserticola polysaccharides	Short-chain fatty acids	^1^H NMR	Rat (fecal)	CUMS	[551]
Cistanche tubulosa	Isobutyrylglycine, citric acid, D-galactose	UPLC-QTOF-MS	Mice (serum, testis)	CUMS	[552]
Saffron essential oil	Dimethylglycine, glycerol, adenosine, β-glucose, α-glucose, uridine, mannose, sarcosine, aspartate	1H NMR	Mice (liver, spleen, kidneys)	CRS	[553]
Gentiopicroside	Arachidonic acid	LC/MS	Rat (hippocampus)	Corticosterone-induced model of depression	[554]
Astragaloside IV	Short-chain fatty acids, amino acids	^1^H NMR	Rat (fecal)	CUMS	[555]
Shuganjieyu capsule	Glutamine, glutamate, arginine	^1^H NMR	patients and healthy controls (plasma)	/	[556]
SheXiangXinTongNing	Tryptophan metabolism, Linoleic acid metabolism	LC/MS	Mice (brian)	CUMS	[543]
Chaihu-Shu-Gan-San	Twenty-six metabolites (16 from the hippocampus and 10 from serum)	UPLC-QTOF-MS	Rat (h		
Ippocampus, serum)	CVS	[557]			
Baihe-Dihuang Tang	d-glutamine, d-glutamate metabolism, purine metabolism, l-glutamate, xanthine, adenine	UHPLC-QTOF-MS	Rat (brain, plasma, urine)	CUMS	[558]
Lily bulb and Rehmannia decoction	Glycerophospholipid metabolism	LC–MS	Rat (prefrontal)	Lipopolysaccharide-induced depression	[559]
Bupleurum chinense DC and Paeonia lactiflora Pall	21 metabolites	UPLC-MS/MS	Rat (serum)	CUMS	[560]
Xiaoyaosan	38 metabolites	^1^H NMR	Rat (serum)	CUMS	[561]
Quercetin	17 metabolites	/	Rat (liver)	CUMS	[562]
Chaigui Granules	Purine metabolism	UPLC-QTOF/MS	Rat (plasma, peripheral blood mononuclear cells)	CUMS	[563]
Icariin	Eight, five and three potential biomarkers associated with depression in serum, urine and brain tissue	^1^H NMR	Rat (serum, urine, brain)	corticosterone-induced depression	[564]
Kai-Xin-San	PC(16:0/P-18:0), PC(16:0/P-18:1(11Z)), PC(O-18:0/20:4(8Z,11Z,14Z,17Z)), PC(P-18:1(11Z)/20:0)	UPLC-QTOF/MS	Depressed patients (serum)	/	[565]
crocin	7 metabolites	LC–MS/MS	Rat (serum)	CUMS	[566]
Platycodins Folium	32 metabolites	UPLC-QTOF-MS	Mice (serum, hippocampus)	Lipopolysaccharide-induced depressive mice	[567]
Xiaoyaosan	Lactic acid, glycerol, glutamine, glutamic acid, hypoxanthine, myo-inositol, cholesterol	GC–MS	Rat (hippocampal)	CUMS	[568]
Xiaoyaosan	Oxalic, stearic acids	GC–MS	17 depressed patients and the 17 healthy subjects (plasma)	/	[569]
Milletia speciosa Champ	10 metabolites	^1^H NMR	Mouse (blood)	CUMS	[570]
Xiaoyaosan	alanine, proline, lactate, valine	^1^H NMR	Rat (cecum)	CUMS	[571]
Yueju Wan	21 metabolites	UPLC-QTOF-MS	Rat (fecal)	CUMS	[572]
Chaihu Shu Gan San	16 metabolites	UPLC-QTOF-MS	Rat (serum)	CUMS	[573]
Changpu San's	42 metabolites	UPLC-QQQ-MS/MS	Mouse (serum)	CUMS	[547]
Xiaoyaosan	Glutamate, malate, taurine	^1^H NMR	Rat (liver)	CUMS	[574]
Xiaoyaosan	Ten metabolites	^1^H NMR	Rat (serum)	CUMS	[575]
Xiaoyaosan	alanine, choline, trimethylamine oxide, glutamine, lactate, glucose	^1^H NMR	Patients (plasma)	/	[576]
xiaoyaosan	13 metabolites	GC–MS	Rat (urine)	CUMS	[577]
Danzhi Xiaoyao Powder	24 metabolites	UPLC-QTOF-MS	Rat (plasma)	CUMS	[578]
Zhi-zi-chi decoction	Seventy six metabolites	UFLC/Q-TOF–MS	Mice (serum)	CUMS	[579]
Millettia speciosa Champ	9 metabolites	UPLC-QTOF-MS	Rat (urine)	CUMS	[580]
Jiaotaiwan	10 metabolites	UPLC-QTOF-MS	Rat (serum)	CUMS	[581]
Xiaoyao San	23 metabolites	^1^H NMR, LC/MS	Rat (liver)	CUMS	[582]
Xiao-Chai-Hu-Tang	9 metabolites	UPLC/MS	Rat (serum)	CUMS	[583]

LC–MS: liquid chromatography mass spectrometry

NMR: nuclear magnetic resonance

UPLC-QTOF-MS: ultra performance liquid chromatography coupled with quadrupole time-of-flight mass spectrometry

UPLC-MS/MS: ultra performance liquid chromatography tandem mass spectrometry

GC–MS: gas chromotography/mass spectrometry

UPLC-QQQ-MS/MS: ultra-high-performance liquid chromatography-tandem quadrupole mass spectrometry

UFLC/Q-TOF–MS: ultra-fast liquid chromatography/quadrupole-time-of-flight tandem mass spectrometry

#### Proteomic association between Chinese medicine and depression targets

##### Screening METHODS FOR ANTIDEPRESSANT TARGETS

In screening antidepressant targets in Chinese medicine, researchers commonly employ a range of methods to identify biomarkers and therapeutic targets associated with depression. In recent years, network pharmacology has emerged as a valuable approach for target identification. By constructing comprehensive “drug–target–disease” interaction networks, researchers can systematically identify potential depression-related targets of Chinese medicine components. For example, by integrating data from the Traditional Chinese Medicine Systems Pharmacology Database (TCMSP) and gene databases such as GeneCards, researchers can extract compound structures, target proteins, and depression-associated genes. Intersection analysis of these datasets allows for the identification of key targets [535, 584, 585].

Additionally, the construction of protein–protein interaction (PPI) networks has further enhanced target screening capabilities. Using platforms like the STRING database, researchers can visualize and analyze interactions among identified targets, enabling the recognition of core targets with high biological relevance. Functional enrichment analyses, such as Gene Ontology (GO) and Kyoto Encyclopedia of Genes and Genomes (KEGG) pathway analyses, further elucidate the biological mechanisms by which Chinese medicine may exert antidepressant effects [535, 586].

In practical research, molecular docking techniques also play a vital role in validating target interactions. By simulating the binding affinity between bioactive compounds and candidate targets, researchers can predict the pharmacological activity of Chinese medicine components. For instance, studies have demonstrated that certain compounds exhibit antidepressant effects through multiple mechanisms, including modulation of neurotransmitter release and inhibition of inflammatory responses [535, 587].

##### Analyses of relevant research

In recent years, numerous studies have investigated the therapeutic targets and mechanisms of Chinese medicine in treating depression by integrating proteomics with experimental validation. For instance, researchers conducted a detailed proteomic analysis of Kaiyu Zhishen Decoction and identified its key active components—primarily flavonoids and phenolic acids—as major contributors to its antidepressant effects. These compounds significantly improved depressive behaviors in mouse models by modulating the MAPK and BDNF signaling pathways [588].

Moreover, studies have shown that specific Chinese medicines, including resveratrol and Xiaoyaosan, alleviate depressive symptoms by regulating multiple biological pathways. These include the ELAVL4-BDNF axis, the HPA axis, and antioxidant defense mechanisms [535, 589]. Such findings provide both theoretical justification and experimental evidence supporting the clinical application of Chinese medicine in depression treatment. The summary of proteomics applications in antidepressant Chinese medicine is presented in Table 9.

**Table 9:** Characteristics of the application of proteomics in antidepressant Chinese medicine

Drug	Biomarkers	Analytical technique	Samples	Model	References
Radix Bupleuri and Radix Paeoniae Alba	Mt1, CDK, UCH-L1	Two-dimensional gel electrophoresis, Liquid phase tandem mass spectrometry	Rat (liver)	CUMS	[590]
Jiawei Danzhi Xiaoyao San	59 differentially expressed proteins	Tandem mass tag (TMT)-based quantitative proteomic analysis	Mouse (brain)	CUMS	[591]
JiaWeiSiNiSan	Rps4x, HSP90AA1, Rps12, Uba1, Rsp14, Tuba1b	Tandem Mass Tag (TMT) proteomics analysis	Rat (cerebrospinal fluid)	CUMS	[592]
Xiaochaihutang	31 differentially expressed proteins	Tandem Mass Tag (TMT) proteomics analysis, LC–MS/MS	Mice (liver)	CUMS	[593]
Shen-Zhi-Ling	alpha-1-antitrypsin, von Willebrand factors, apolipoprotein C-III, alpha-2-macroglobulin	Label-free quantitative proteomic analysis, liquid chromatography-tandem mass spectrometry	Patients (serum)	/	[594]
Erxian Decoction	2620 proteins	Data independent acquisition (DIA)-mass spectrometry (MS)	Rat (cerebrospinal fluid)	CUMS	[595]
Sinisan	82 differentially expressed proteins	Tandem mass tag (TMT) based quantitative proteomics analysis	Mouse (liver)	CUMS	[596]
Kaiyu Zhishen decoction	103 differentially expressed proteins	4D-DIA proteomic sequencing	Mice (hippocampal)	CUMS	[588]
Kai-Xin-San	33 differentially expressed proteins	iTRAQ, LC–MS	Rat (hippocampus)	CMS	[597]
Chaihu-Shugan-San	407 differentially expressed proteins	TMT Labeled Quantitative Proteomics	Rat (hippocampus)	CUMS	[598]
Luteolin	EFNA5, EPHB4, EPHA4, SEMA7A, NTNG, UNC5B, L1CAM, DCC	Data Independent Acquisition (DIA) Method	Rat (cerebrospinal fluid)	CUMS	[599]
Xiao Yao San	CORT, UCN2	Two-Dimensional Electrophoresis (2D) and Protein Identification	Rat (hippocampus)	CUMS	[589]

iTRAQ: isobaric tags for relative and absolute quantitation

LC–MS/MS: liquid chromatography-tandem mass spectrometry

LC–MS: liquid chromatography mass spectrometry

#### Microbiome as a target for antidepressant chinese medicine

##### Application of microbiomics in antidepressant Chinese medicine targets

The microbiome has increasingly emerged as a key target in the study of antidepressant Chinese medicine. Recent research highlights the strong association between gut microbiota composition and the onset and progression of depression. An imbalance in the microbiome is now recognized as a significant factor contributing to depression. In the context of Chinese medicine, scientists are exploring how to alleviate depressive symptoms by modulating the microbiome. For example, Chinese formulations such as Xiaoyaosan and Danzhi-Xiaoyao-San have demonstrated the ability to regulate gut microbiota in animal models, leading to improved depressive behaviors. The active ingredients in these formulations may influence the host's physiological and psychological states by altering the composition of the gut microbiome.

Research has demonstrated that the diversity of the gut microbiota in depression model rats treated with Xiaoyaosan was significantly increased. Additionally, the abundance of beneficial bacteria, such as lactic acid bacteria, was notably enhanced, which correlated with the alleviation of depressive symptoms [571]. Similarly, studies on Danzhi-Xiaoyao-San indicate that it exerts an antidepressant effect by modulating the composition of the gut microbiota in patients with depression and influencing metabolic pathways [600].

By integrating high-throughput sequencing with metabolomics, researchers can more comprehensively explore how Chinese medicine components influence the host's physiological and psychological states through microbiome regulation. For example, studies have shown that certain components of Chinese medicine can improve depressive symptoms by modulating the metabolites of gut microbiota, such as the hydrolysis of fatty acid amides, which in turn influences host neurotransmitter levels [601]. These studies not only offer new insights into the mechanisms of antidepressant Chinese medicines but also highlight potential targets for future drug development.

##### Development of new drugs through microbial target development

Chinese medicine, with its unique ability to regulate the microbiome, presents a promising avenue for such drug development. Studies have shown that components of Chinese medicine not only directly influence neurotransmitter balance but also indirectly affect the onset of depression by modulating the composition and function of the gut microbiota [602–605].

For example, animal model studies have demonstrated that treatment with Xiaoyaosan for depression leads to changes in the gut microbiota that correlate with improvements in depressive symptoms. By modulating the microbiome, these Chinese medicines promote the growth of beneficial bacteria while inhibiting the proliferation of harmful bacteria, ultimately enhancing the host's psychological state [606]. Additionally, research on Danzhi-Xiaoyao-San has revealed how it affects neurotransmitter levels by altering the metabolites of the intestinal microbiota, offering new insights for developing future therapeutic drugs [600].

In the development of new drugs, combining microbiome-targeted therapies with a multi-target, multi-pathway approach can enhance therapeutic efficacy while reducing side effects. This strategy not only improves drug effectiveness but also minimizes potential adverse effects. For example, studies have demonstrated that certain components of Chinese medicine alleviate depressive symptoms by regulating intestinal microbiota metabolites, which may also serve as targets for new drug development [607]. By further investigating the relationship between the microbiome and depression and integrating modern drug development techniques, we anticipate the creation of more effective antidepressant treatments in the future. A summary of the role of microbiomics in antidepressant Chinese medicine is presented in Table 10.

**Table 10:** Characteristics of the application of microbiomics in antidepressant Chinese medicine

Drug	Biomarkers	Analytical technique	Samples	Model	References
Puerarin	Proteobacteria, Flexispira, Desulfovibrio, Firmicutes, Bacillales, Lactobacillus	16S rRNA sequencing	Mice (fecal)	CUMS	[608]
Xiaoyaosan	Peptostreptococcaceae	16S rRNA sequencing	Rat (fecal)	CUMS	[550]
Cistanche deserticola polysaccharides	Bacteroides, Parabacteroides, Blautia	16S rRNA sequencing	Rat (fecal)	CUMS	[551]
Astragaloside IV	Lactobacillus and Oscillospira	16S rRNA sequencing	Rat (fecal)	CUMS	[555]
SheXiangXinTongNing	Alloprevotella, Helicobacter, Allobaculum, Clostridia	16S rRNA sequencing	Mice (fecal)	CUMS	[543]
Yueju Wan	Eubacterium, Oscillibacter, Roseburia, Romboutsia, Bacterium	Metagenomics sequencing analysis	Rat (fecal)	CUMS	[572]
Xiaoyaosan	Bacteroidetes, Proteobacteria, Firmicutes, Chloroflexi, Planctomycetes, Prevotellaceae_Ga6A1_group, Prevotellaceae_UCG-001, Desulfovibrio,	16 S rDNA sequencing	Rat (fecal)	Chronic immobilization stress	[609]
Xiaoyaosan	31 potential microbial	16S rRNA sequencing	rat (fecal)	CUMS	[610]
Crocin-I	Proteobacteria, Bacteroidetes, Sutterella spp., Ruminococcus spp., Firmicutes, Lactobacillus spp., Bacteroides spp	16S rRNA sequencing	Mice (fecal)	CRS	[611]
Jasmine Tea	Patescibacteria, Firmicutes, Bacteroidetes, Spirochaetes, Elusimicrobia, Proteobacteria	16S rRNA sequencing	Rat (fecal)	CUMS	[612]
Schisandrin	Bacteroidetes, Firmicutes, Bacteroidia, Clostridia, Lactobacillaceae, Barnesiella, Alloprevotella, Lactobacillus	16S rRNA sequencing	Mice (fecal)	LPS-induced depressive-like behaviors	[613]
Sophora alopecuroides L	Lactobacillus, Helicobacter, Oscillospira, Odoribacter, Mucispirillum, Ruminococcus	16S rRNA sequencing	Mice (fecal)	CUMS	[614]
Saikosaponin A	Firmicutes, Verrucomicrobia	16S rRNA sequencing	Mice (fecal)	Reserpine-induced depressive-like symptoms	[615]
Dihydroartemisinin	Turicibacter, Lachnospiraceae, Erysipelotrichaceae, Erysipelatoclostridium, Eubacterium, Psychrobacter, Atopostipes, Ileibacterium, Coriobacteriacea, Alistipes, Roseburia, Rikenella, Eggerthellaceae, Ruminococcus, Tyzzerella, Clostridia	16S rRNA sequencing	Mice (fecal)	CUMS	[616]
Gastrodia elata Blume water extract	Actinobacteria	16 S rDNA sequencing	Mouse (fecal)	CSDS	[617]
Jasmine tea extract	Romboutsia, Blautia, Monoglobus, Bifidobacterium, Clostridium_sensu_stricto_1, Escherichia-Shigella	16S rRNA gene sequencing	Rat (fecal)	CUMS	[618]
Cryptotanshinone	Paraacteroides distasonis, Paraacteroides Merdae, Pseudomonas Caeni	16 S rDNA sequencing	Rat (fecal)	CUMS	[619]
Xiaoyao Pills	Benzoic acid, liquiritigenin, glycyrrhetinic acid, saikogenin D	16S rRNA gene sequencing	Rat (fecal)	CUMS	[601]
Xiaoyaosan	Bacteroides, Corynebacterium, Lactobacillus, Adlercreutzia	16S rRNA gene sequencing	Rat (fecal)	CUMS	[620]
Paeonia lactiflora Pall. Polysaccharide	Proteobacteria, Bacteroidetes, Actinobacteria, uncultured Bacteroidales bacterium, unclassified Muribaculaceae, Parasutterella, Ligilactobacillus, Klebsiella	16S rRNA gene sequencing	Mice (fecal)	CUMS	[621]
Shugan granule	Bacteroides, Butyricimonas, Candidatus Arthromitus	16S rDNA sequencing	Rat (fecal)	CRS	[622]
Leonurine	Lactobacillus, Lachnospiraceae_NK4A136_group, Clostridia_UCG-014, Prevotellaceae_Ga6A1_group	16S rRNA sequencing	Rat (fecal)	CUMS	[623]
Gegen Qinlian Decoction	Ruminococcus, Lachnoclostridium, Pygmaiobacter, Bacteroides, Pseudomonas, Pseudomonas Family_XIII_AD3011_group	16S rRNA sequencing	Rat (fecal)	CUMS	[624]
Eucommiae cortex polysaccharides	Lactobacillaceae	16S rRNA sequencing	Mice (fecal)	CUMS	[625]
Kai-Xin-San	Allobaculum, Bifidobacterium, Turicibacter, Coprococcus, Helicobacter, Mucispirillum, Odoribacter, Oscillospira	16S rRNA sequencing	Mice (fecal)	CUMS	[626]
Arecoline	Bifidobacterium pseudolongum, Ligilactobacillus murinus	16S rRNA sequencing	Mice (fecal)	CUMS	[627]
Xiaoyaosan	Firmicutes, Actinobacteria	16S rRNA sequencing	Rat (fecal)	CUMS	[571]

### Specificity note

**Fig. 2:**
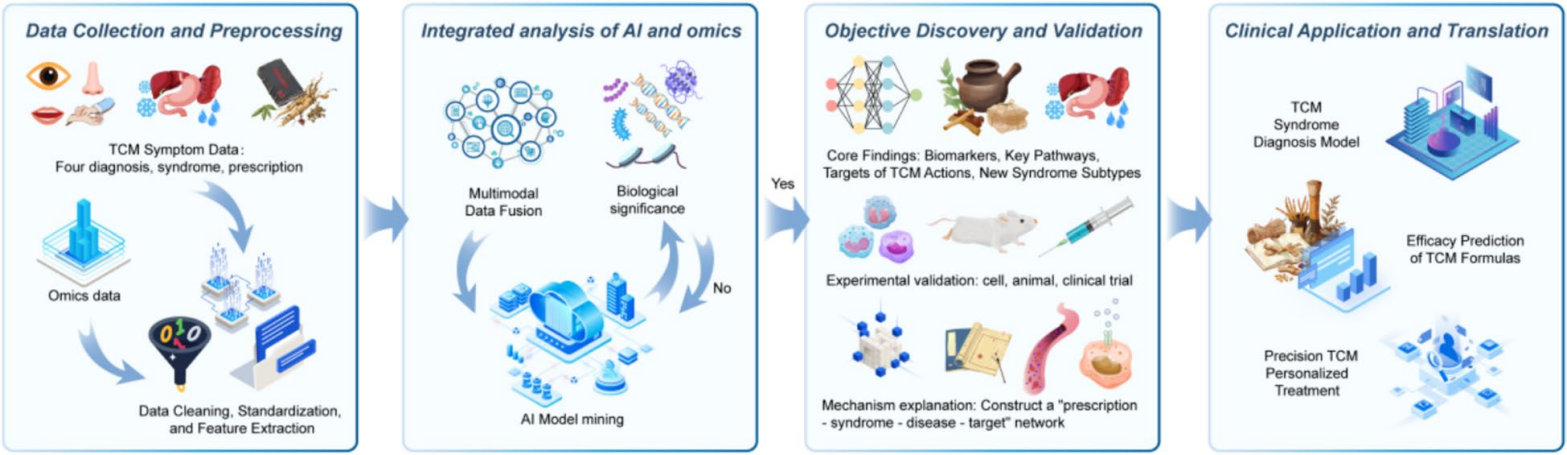
A flowchart of the combined approach of AI and omics in the discovery of research goals in traditional Chinese medicine. TCM Syndrome Diagnosis Model: Develop syndrome waiting diagnosis kits or software based on biomarkers. Efficacy Prediction of TCM Formulas: Establish an AI platform to predict a patient's response to a specific prescription and assist in clinical decision-making. Precision TCM Personalized Treatment: Based on the patient's molecular typing and TCM constitution, a tailored treatment plan integrating traditional Chinese and Western medicine is created for each individual

Some of the biomarkers we summarized not only show reasonable specificity for the pathophysiology and clinical improvement of depression. More importantly, the dynamic changes of biomarkers are closely related to the clinical efficacy of traditional Chinese medicine treatment, providing a modern scientific basis for the "syndrome differentiation and treatment" and "overall regulation" of traditional Chinese medicine. DNA methylation is one of the most significant epigenetic modifications associated with depression, and DNA methyltransferase (DNMT) is a key enzyme involved in this process. DNMT may help improve the cognitive and emotional domains of depressive phenotypes, and the mRNA level of DNMT1 in the peripheral blood of patients with episodic depression is decreased [1, 628]. Studies have shown that Xiaoyao Pill upregulates the expression of DNMT1 in patients with depression, salvaging the abnormal gene expression and DNA methylation patterns associated with depression [2, 526]. Lmx1b is crucial for the development and differentiation of midbrain dopaminergic neurons, and it is also essential for the differentiation and survival of central serotonin neurons during embryonic development [3, 4, 629, 630]. Xiaoyaosan effectively reverses depression-like behaviors. The loci-specific methylation levels and regulation of Lmx1b, Abcc5, Gpc3, and Cfb genes may play a key role in the treatment of Xiaoyaosan [5, 531]. Metabolic profiling, as an "overall model", has high specificity and can distinguish patient populations from healthy populations with considerable accuracy. Oxalic and stearic acids, as metabolic biomarkers for the early diagnosis of patients with depression, can not only integrate the sensitivity and specificity of individual biomarkers, but also improve the overall diagnostic rate [6, 569]. Researchers found that, compared with healthy controls, the levels of trimethylamine oxide, glutamine, and lactic acid in the plasma of patients with depression were significantly increased, while the levels of phenylalanine, valine, alanine, glycine, leucine, citrate, choline, lipids, and glucose were significantly decreased. After Xiaoyaosan's treatment, alanine, choline, trimethylamine oxide, glutamine, lactic acid, and glucose returned to normal levels [7, 576]. Similar to metabolites, proteins, as the direct executors of life functions, have demonstrated outstanding potential and unique specificity in proteomic research on depression. Proteomics can also effectively distinguish patients with depression from healthy controls. For instance, by analyzing the blood of patients with depression and healthy controls, a "profile" composed of 12 proteins can be identified. It has been verified and confirmed that Shen-zhi-Ling can simultaneously regulate the expression of alpha-1-antitrypsin, von Willebrand factors, apolipoprotein C-III, and alpha-2-macroglobulin [8, 594]. Unlike traditional pharmacology, which studies a single compound or a single target, omics can capture the global effects of traditional Chinese medicine from a systematic perspective. This "systematic" approach precisely aligns with the "holistic view" of traditional Chinese medicine. The process of discovering research targets in traditional Chinese medicine by combining AI and omics is shown in Fig. 2.

## Prospects for the integration of AI and omics

### Technological development trends

The rapid advancement of AI and omics technologies is reshaping medical research. AI—particularly machine learning and deep learning—has demonstrated exceptional capabilities in processing complex datasets, making it especially valuable for analyzing multi-omics data. In recent years, biomedical researchers have increasingly applied AI to interpret vast volumes of omics information, including genomics, transcriptomics, proteomics, and metabolomics, to uncover disease mechanisms and identify potential biomarkers. Studies have shown that AI can detect biomarker signatures associated with depression by analyzing large-scale datasets, thus supporting the development of personalized treatment strategies [631–634]. For instance, machine learning algorithms can analyze genomic data to pinpoint genetic variants linked to depression, offering precise clinical guidance [635–638]. The integration of AI and omics has also propelled advances in network pharmacology. This approach enables researchers to explore the mechanisms of traditional Chinese medicine through the lens of systems biology, thereby enhancing both the efficiency and accuracy of drug discovery and development [247, 639–641].

Continued technological progress will expand the role of AI in omics research. AI will be particularly instrumental in data integration and analysis, where optimized algorithms and advanced models can improve our understanding of complex biological networks and accelerate the growth of precision medicine. For example, AI-driven methods for multi-omics integration can better capture disease heterogeneity and inform clinical decision-making [642, 643]. At the same time, as data acquisition technologies evolve, the scale and complexity of omics datasets will grow, placing greater demands on the performance and adaptability of AI algorithms.

### Impact on clinical application

The integration of AI and omics technologies not only advances basic research but also has significant clinical implications. By leveraging AI, clinicians can analyze patients’ omics data more rapidly and accurately, enabling personalized medical care. For example, AI can track real-time changes in patients’ biomarkers and promptly adjust treatment strategies, thereby improving survival rates and quality of life [251, 644–646]. In the field of Chinese medicine, combining AI with omics offers innovative approaches for target discovery and mechanistic research. Multi-omics analyses of Chinese medicine components allow researchers to identify potential biological targets and elucidate mechanisms of action, laying a scientific foundation for its modernization and global integration [639, 647, 648]. This approach not only increases the efficiency of research and development but also enhances the international competitiveness of Chinese medicine.

Despite its promise, applying AI in clinical settings still presents challenges. Key issues include data quality and standardization, the interpretability of AI algorithms, and ethical and legal concerns. To allow broad clinical adoption, the field should establish unified data standards and a shared platform to facilitate multidisciplinary collaboration and communication [649–651].

### Integrated research on comprehensive omics

Multi-omics data integration can be classified into early, middle, and late stages based on the time of integration in analysis and interpretation. In the early integration stage, features from different data matrices are concatenated, but this method faces challenges related to high dimensionality and a small sample size. The mid-term integration strategy involves transforming each omics dataset into a simpler representation through dimensionality reduction, and then integrating the merged datasets through machine learning without concatenating features or merging results. Late integration is the result of combining various multi-omics datasets after independent analysis at each omics level [1, 652].

Multi-omics integration methods are classified by "learning type + integration type". Supervised learning includes series-based methods such as decision trees, NB, SVM, RF, and other classic algorithms, model-based methods such as majority voting, XGBoost, MOLI, and transformation-based methods including SDP-SVM and FSMKL. Unsupervised learning includes concatenation-based methods such as Joint NMF and iCluster, model-based methods such as PSDF and SNF, and transform-based methods such as rMKL-LPP and NEMO. This provides a direct reference for the selection of integration methods [2, 653].

Spatial multi-omics technology has significantly enhanced our understanding of biological systems by providing molecular maps with spatial resolution capabilities across multiple levels. Existing spatial multi-omics integration methods typically assume that data of different modalities share a common latent distribution, aiming to project them into a unified low-dimensional space. However, this assumption may obscure the unique biological information inherent in each mode, thereby limiting the comprehensiveness of multi-omics analysis. To overcome this limitation, researchers have proposed the Spatial Multi-View (SpaMV) representation learning algorithm. This method can simultaneously capture common information among different modalities as well as unique information specific to each modality, thereby achieving a more comprehensive and interpretable representation of spatial multi-omics data. Through systematic evaluation of both simulated and real data, SpaMV demonstrated superior performance in spatial domain clustering tasks and provided more biologically interpretable dimensionality reduction results for downstream analysis [3, 654].

**Fig. 3:**
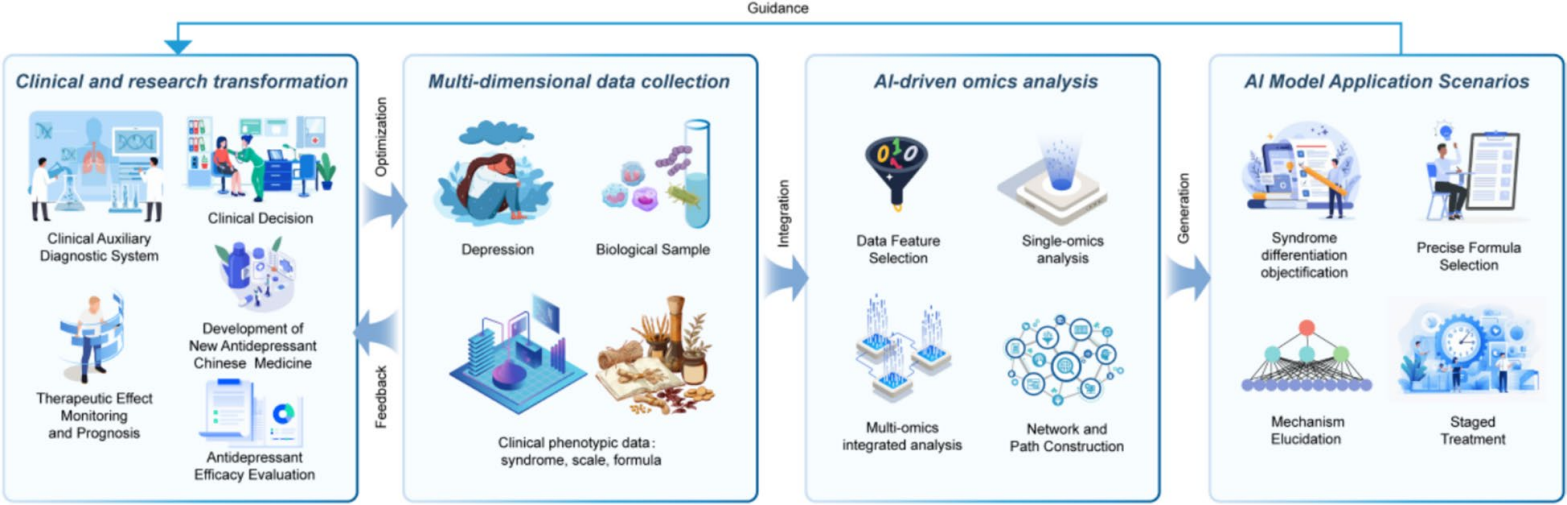
Schematic diagram of the specific application of AI combined with omics in the prevention and treatment of depression with traditional Chinese medicine

From traditional multi-omics association analysis to machine learning-driven biomarker mining, and then to deep learning-led multi-modal deep integration, we are witnessing a profound transformation in the research paradigm of life sciences. The core thread of this technological evolution path is from "description" to "prediction", and ultimately towards "understanding" (Fig. 3).

## Challenges and future directions

### Data sharing and standardization issues

Data sharing and standardization remain major challenges in applying AI and omics technologies to Chinese medicine research. Although AI can efficiently process and analyze large-scale omics data, the diversity and complexity of data sources hinder standardization and data sharing. Differences in experimental methods, data formats, and recording practices across research institutions create significant barriers to cross-institutional data integration [639, 655]. Moreover, the absence of unified data standards contributes to low data reuse rates, ultimately reducing the efficiency and reliability of research outcomes.

To address these issues, researchers must develop unified data standards and establish shared platforms to enhance data interoperability and reproducibility. For example, in network pharmacology, researchers are working to build web-based AI tools to uncover therapeutic mechanisms of complex diseases. These efforts aim to improve the comparability and reliability of findings through the adoption of standardized data formats [245, 647]. In parallel, government agencies and research institutions should promote policies that support data standardization and sharing, ensuring a collaborative and scalable research environment.

### Interpretability of AI models

The interpretability of AI models presents another critical challenge. While AI technologies, particularly deep learning, have shown impressive predictive power in Chinese medicine research, their "black box" nature makes it difficult for researchers to understand the model’s decision-making process [656, 657]. This lack of transparency undermines trust in the model’s results and limits its adoption in clinical practice.

To address this issue, researchers are exploring Explainable AI (XAI) techniques to enhance model transparency and interpretability. For instance, by applying visualization methods and feature importance analysis, researchers can gain insights into how the model makes decisions, thereby increasing trust in its outcomes [658–660]. Future research must prioritize improving the interpretability of AI models to facilitate their broader application in Chinese medicine research and ensure their effectiveness and safety in clinical decision-making.

### Ethical and regulatory issues of artificial intelligence

Ethical concerns are a critical aspect of integrating AI and omics in research, particularly in the context of depression. As AI becomes increasingly prevalent in both research and clinical applications, a range of ethical and regulatory challenges has emerged. Key issues include protecting patient privacy, ensuring data security, and addressing algorithmic bias. The use of AI in mental health raises particular concerns around data privacy, fairness, and patient autonomy [661]. For example, AI systems that process sensitive personal health data may infringe on patients' privacy rights if transparency and regulatory oversight are lacking [662]. To address these concerns, future research must prioritize the development of robust ethical frameworks and regulatory guidelines for AI applications. Collaboration between researchers and policymakers is essential to safeguard patient rights and ensure the safe, responsible, and sustainable use of AI technologies [663–667]. Additionally, it is crucial to improve clinicians’ understanding and practical use of AI through targeted education and training. Building this competence will support the effective integration of AI into depression management and enhance clinical decision-making [668, 669].

### Development directions for future research

The integration of AI and omics has opened unprecedented opportunities for both medical research and clinical applications. However, to fully harness its potential, further research is required in key areas such as data integration, model interpretability, and ethical governance. With sustained effort, this integration will provide robust support for the advancement of precision medicine.

Future research should prioritize several critical directions. First, promoting data sharing and implementing standardized protocols will improve the efficiency and reliability of Chinese medicine research. Second, enhancing the interpretability of AI models is essential so that researchers and clinicians can understand model decision-making processes and apply these technologies more effectively in practice. In parallel, relevant institutions must establish clear policies to address ethical concerns surrounding AI applications. Researchers should also deepen the integration of AI with TCM methodologies to generate more comprehensive and scientifically grounded findings. For example, combining AI with network pharmacology can help elucidate the multi-target mechanisms of Chinese medicine in greater detail [670].

Finally, as technology continues to evolve, future studies should explore the application of emerging tools in TCM research. These may include blockchain-based platforms for secure data sharing or big data analytics to uncover the latent therapeutic value of Chinese medicine. Such efforts will accelerate the scientific development of TCM and provide a stronger, evidence-based foundation for clinical applications [671].

## Conclusion

The rapid advancement of AI and omics technologies has created unprecedented opportunities for identifying therapeutic targets in antidepressant Chinese medicine. This interdisciplinary and integrated research model not only accelerates the modernization of TCM but also introduces a novel framework for the precision treatment of depression. Whereas earlier research primarily focused on traditional pharmacology and clinical studies, the integration of AI and omics now allows researchers to explore the mechanisms of Chinese medicine from a broader systems-level perspective, enabling more effective identification and validation of potential targets.

One of the main challenges in this field lies in reconciling the diverse findings and perspectives of existing studies. Although research has demonstrated the therapeutic potential of Chinese medicine in treating depression, inconsistencies in experimental design, sample selection, and data analysis methods have led to significant heterogeneity in results. AI technologies can address these limitations by standardizing data processing and overcoming the constraints of conventional research approaches. Using data mining and pattern recognition techniques, AI can extract clinically meaningful targets from complex datasets. For instance, machine learning algorithms can identify depression-related biomarkers within large-scale omics data, offering a scientific foundation for the development of targeted therapies based on Chinese medicine [671, 672].

Despite the significant promise of combining AI and omics technology, this approach has sparked some controversy. Critics have questioned its accuracy and reproducibility, expressing concerns that excessive reliance on algorithms might lead to a narrow or oversimplified understanding of the complexity inherent in Chinese medicine [673, 674]. As a result, future research should emphasize interdisciplinary collaboration, foster communication between clinical and experimental research, and ensure that the insights generated by AI and omics technologies are both verifiable and applicable in clinical settings.

Fundamental discoveries driven by AI and omics drive the generation of high-quality, verifiable hypotheses. Preclinical studies have confirmed the functional effects and pharmacological correlations of candidate targets for treating depression with traditional Chinese medicine. Further analyze the diagnostic value, predictive value, and therapeutic effectiveness of biomarkers and traditional Chinese medicines for depression through standardized clinical trials. Moreover, we can simplify complex multi-group biological markers into standardized tests that can be implemented in clinical laboratories (such as a Western blot for protein detection or qPCR for gene detection). By collaborating with in vitro diagnostic companies, we can develop, optimize, and validate the detection methods to ensure their reliability, sensitivity, and specificity. In the later stage, validated diagnostic tools and treatment methods were used in a broader and more complex group of real patients with depression, and their effectiveness and safety were observed for a long time. Additionally, the verified AI prediction models and biomarker panels are integrated into the hospital's information system, providing doctors with decision support. For instance, the system automatically reads the patient's test results and prompts: "This patient has' liver depression and spleen deficiency 'depression. It is recommended to try Xiaoyaosan with modifications. The expected effective rate is 85%.". Collect new data and feedback from real-world applications to feed back and optimize the initial AI model, forming a closed-loop learning system that continuously evolves the entire system and promotes the precise treatment of traditional Chinese medicine for depression.

However, our work also has certain limitations. Firstly, the manuscript lists AI algorithms (such as machine learning and deep learning), but does not delve deeply into which algorithm is most suitable for solving the specific problem of "identifying key driver targets from a large amount of omics data". What AI models learn from data are mostly statistical correlations rather than causal correlations. Our exploration of cutting-edge methods, such as causal inference and multi-omics fusion, may be insufficient. Secondly, the advantage of traditional Chinese medicine lies in its systematic regulation. Its effect is often achieved by regulating the network formed by multiple targets rather than inhibiting or activating a single target. The traditional paradigm of "one drug, one target" may not be applicable when evaluating traditional Chinese medicine. The "key targets" predicted by AI may not accurately capture the essence of this network regulation. Finally, this article may overly emphasize the technical capabilities of AI and metabolomics, while lacking depth in exploring the fundamental challenges currently existing, such as the quality of omics data and the complexity of drug mechanisms. Furthermore, the manuscript did not establish a comprehensive closed-loop verification framework, encompassing "computational prediction—in vitro validation—in vivo functional confirmation—clinical relevance analysis." Therefore, this is a promising but bumpy track. Its success ultimately depends on the accumulation of high-quality standardized data, the close closed-loop collaboration between bioinformatics and experimental biology, as well as the creative resolution of the contradiction between the holistic treatment philosophy of TCM and the discovery of reductionist targets.

Overall, the integration of AI and omics technologies has established a strong foundation for identifying therapeutic targets in antidepressant Chinese medicine. Future research should continue to deepen this interdisciplinary approach, promote the convergence of diverse perspectives, and drive ongoing advancements in depression treatment. This approach not only represents an active effort to modernize Chinese medicine but also holds the key to providing hope for countless individuals suffering from depression.

## Data Availability

No datasets were generated or analysed during the current study.

## Abbreviations

Abcc5: ABC transporter C family member 5
ADAMTS5: A disintegrin and metalloproteinase with thrombospondin motifs 5
Adcyap1R1: Pituitary adenylate cyclase-activating polypeptide type I receptor
AHR: Aryl hydrocarbon receptor
ANGPTL3: Angiopoietin-related protein 3
AI: Artificial Intelligence
AUC: Area Under the ROC curve
ApoA-IV: Apolipoprotein A-IV
ApoC-II: Apolipoprotein C-II
APOE: Apolipoprotein E
APOH: Apolipoprotein H
ATIII: Antithrombin-III
BAG5: BAG family molecular chaperone regulator 5
BDNF: Brain-derived neurotrophic factor
BERT: Bidirectional Encoder Representations from Transformers
BHLHE22: Class E basic helix-loop-helix protein 22
BTN3A3: Butyrophilin subfamily 3 member A3
CAMK2A: Calcium/calmodulin-dependent protein kinase type II subunit alpha
CARTPT: Cocaine- and amphetamine-regulated transcript protein
CCK: Cholecystokinin
CDK: Cyclin-dependent kinase
Cfb: Complement factor B
CFI: Complement factor I
CGANs: Conditional Generative Adversarial Networks
CpG: Cytosine-phosphate-Guanine
CpGs: Cytosine-phosphate-Guanines
CLEC4A: C-type lectin domain family 4 member A
CMS: chronic mild stressed
CNNs: convolutional neural networks
CNTNAP4: Contactin-associated protein-like 4
COL1A2: Collagen alpha-2(I) chain
CORT: corticosterone
COX5B: Cytochrome c oxidase subunit 5B
CREB: cAMP response element-binding protein
CRP: C-reactive protein
CRS: chronic restraint stress
CSDS: chronic social defeated stress
CUMS: chronic unpredictable mild stress
CVS: chronic variable stress
DBNL: Drebrin-like protein
DBN1: Drebrin
DEFB1: Beta-defensin 1
DEGs: differentially expressed genes
DIA: Data Independent Acquisition
DNMT: DNA methyltransferase
DNMT1: DNA (cytosine-5)-methyltransferase 1
DRL: deep reinforcement learning
ECG: electrocardiogram
EFNA5: Ephrin-A5
EGF: Epidermal growth factor
EHRs: electronic health records
EPHA4: Ephrin type-A receptor 4
EPHB4: Ephrin type-B receptor 4
ESR1: Estrogen Receptor 1
ESR2: Estrogen Receptor 2
EWAS: epigenome-wide association study
FGFR1: Fibroblast growth factor receptor 1
FGF21: Fibroblast growth factor 21
FISH: fluorescence in situ hybridization
FNDC3B: Fibronectin type III domain-containing protein 3B
FOXO1: Forkhead box protein O1
FSMKL: Multiple Kernel Learning with Feature Selection
GANs: generative adversarial networks
GAPDH: Glyceraldehyde-3-phosphate dehydrogenase
GC: gas chromatography
GC-MS: gas chromotography/mass spectrometry
GEO: Gene Expression Omnibus
GHSR: Growth hormone secretagogue receptor type 1
GO: Gene Ontology
Gpc3: Glypican-3
GPR173: Probable G-protein coupled receptor 173
GPT: Generative Pre-trained Transformer
GRIA4: Glutamate receptor 4
GRUs: Gated Recurrent Units
GWAMA: genome-wide association meta-analyses
GWAS: genome wide association study
HACE1: HECT Domain and Ankyrin Repeat Containing E3 Ubiquitin Protein Ligase 1
HAMD: Hamilton Depression Scale
HBA: Hemoglobin subunit alpha
HBEGF: Heparin-binding EGF-like growth factor
HILIC-MS: liquid chromatography-mass spectrometry
HOMER1: Homer protein homolog 1
HPA: hypothalamic-pituitary-adrenal
HPLC-MS: high performance liquid chromatography and mass spectrum
HSP90AA1: Heat shock protein HSP 90-alpha
HTS: high-throughput screening
IGFBP1: Insulin-like growth factor-binding protein 1
IL-1β: interleukin-1β
IL-6: interleukin-6
IL8: Interleukin-8
ISS: in situ sequencing
ITIH1: Inter-alpha-trypsin inhibitor heavy chain H1
ITIH4: Inter-alpha-trypsin inhibitor heavy chain H4
iTRAQ: isobaric tags for relative and absolute quantitation
KEGG: Kyoto Encyclopedia of Genes and Genomes
KRT23: Keratin, type I cytoskeletal 23
LC: liquid chromatography
LC-MS: liquid chromatography mass spectrometry
LC-MS/MS: liquid chromatography-tandem mass spectrometry
LC-TOFMS: LC-time-of-flight mass spectrometry
LHPP: Phospholysine phosphohistidine inorganic pyrophosphate phosphatase
LIN28B: Protein lin-28 homolog B
LLD: late-life depression
Lmx1b: LIM homeobox transcription factor 1-beta
LRFN5: Leucine-rich repeat and fibronectin type-III domain-containing protein 5
LRG: Leucine-rich alpha-2-glycoprotein
LSTM: Long Short-Term Memory
LSTMs: Long Short-Term Memorys
L1CAM: Neural cell adhesion molecule L1
L3MBTL2: Lethal(3)malignant brain tumor-like protein 2
MALDI-TOF MS: matrix-assisted laser desorption ionization time of flight mass spectrometry
MALDI-TOF-MS/MS: matrix-assisted laser desorption ionization-time of flight-tandem mass spectrometry
MALDI-TOF/TOF MS: matrix-assisted laser desorption/ionization-time-of-flight/time-of-flight tandem mass spectrometry
MAOIs: monoamine oxidase inhibitors
MAPK1: Mitogen-activated protein kinase 1
MDD: major depressive disorder
MEF2C: Myocyte-specific enhancer factor 2C
MHC: Myosin heavy chain
MIF: Macrophage migration inhibitory factor
MLC1: megalencephalic leukoencephalopathy with subcortical cysts 1
MLF1: Myeloid leukemia factor 1
MMP-9: Matrix metalloproteinase-9
MOLI: Multi‐Omics Factor Analysis
MPC: model predictive control
MPO: Myeloperoxidase
MRM-MS: targeted proteomic approach, Multiple reaction monitoring-mass spectrometry
MRPL46: Large ribosomal subunit protein mL46
MS: mass spectrometry
Mt1: Metallothionein-1
MWAS: Methylome-wide association study
MYH9: Myosin-9
MYO16: Unconventional myosin-XVI
MYRF: Myelin regulatory factor
NB: Naive Bayes
NCAM1: Neural cell adhesion molecule 1
NCOA2: Nuclear receptor coactivator 2
NECAB2: N-terminal EF-hand calcium-binding protein 2
NEGR1: Neuronal growth regulator 1
NEMO: NEighborhood based Multi-Omics clustering
NF-κB: nuclear factor κB
NGS: next-generation sequencing
NLP: natural language processing
NMF: Non-negative Matrix Factorisation
NMR: nuclear magnetic resonance
NMT: neural machine translation
NPY: Pro-neuropeptide Y
NRCAM: Neuronal cell adhesion molecule
NRXN3: Neurexin-3-beta
NTRK3: NT-3 growth factor receptor
OGDH: 2-oxoglutarate dehydrogenase complex component E1
OLFM4: Olfactomedin-4
PACAP: pituitary adenylate cyclase-activating polypeptide
PCA: Principal Component Analysis
PDCD6IP: Programmed cell death 6-interacting protein
PDE3A: cGMP-inhibited 3',5'-cyclic phosphodiesterase 3A
PFC: prefrontal cortex
PFN1: Profilin-1
PHLD: Phloroglucinol synthase
PHQ-9: Patient Health Questionaire-9
PLSCR1: Phospholipid scramblase 1
PPARA: Peroxisome proliferator-activated receptor alpha
PPARGC1A: Peroxisome proliferator-activated receptor gamma coactivator 1-alpha
PPT: 20(S)-protopanaxatriol
PPTC7: Protein phosphatase PTC7 homolog
PPI: protein–protein interaction
PRDM1: PR domain zinc finger protein 1
PRDX2: Peroxiredoxin-2
PRKACA: cAMP-dependent protein kinase catalytic subunit alpha
PRKRA: Interferon-inducible double-stranded RNA-dependent protein kinase activator A
PROK2: Prokineticin-2
PROX2: Prospero homeobox protein 2
PRS: polygenic risk scores
PSDF: Patient-Specific Data Fusion
PSMB4: Proteasome subunit beta type-4
PYHIN1: Pyrin and HIN domain-containing protein 1
P2RX7: P2X purinoceptor 7
qPCR: Quantitative real-time polymerase chain reaction
OR8B4: Olfactory receptor 8B4
R-CNN: Region-based Convolutional Neural Network
REST: RE1-silencing transcription factor
RF: random forest
RGS2: Regulator of G-protein signaling 2
rMKL-LPP: regularised multiple kernel learning for Locality Preserving Projections
RNF150: RING finger protein 150
RNNs: recurrent neural networks
ROBO1: Roundabout homolog 1
RPLC-MS: reversed-phase liquid chromatography-mass spectrometry
RPN: Region Proposal Network
Rps12: Small ribosomal subunit protein eS12
Rps4x: Small ribosomal subunit protein eS4
RTN1: Reticulon-1
RUNX1: Runt-related transcription factor 1
SAA1: Serum amyloid A-1 protein
SCFAs: short-chain fatty acids
SCN9A: Sodium channel protein type 9 subunit alpha
SDP-SVM: Semi-Definite Programming SVM
SEMA7A: Semaphorin-7A
SERPINH1: Serpin H1
SDHA: Succinate dehydrogenase [ubiquinone] flavoprotein subunit, mitochondrial
SHANK2: SH3 and multiple ankyrin repeat domains protein 2
SLC12A5: Solute carrier family 12 member 5
SLC6A15: Sodium-dependent neutral amino acid transporter B(0)AT2
SLC6A4: Sodium-dependent serotonin transporter
SLC7A8: Large neutral amino acids transporter small subunit 2
SLIT2: Slit homolog 2 protein
SMS: shotgun metagenomics sequencing
SNF: Similarity Network Fusion
SNPs: single nucleotide polymorphisms
SNVs: whole-genome single nucleotide variants
SpaMV: Spatial Multi-View
SPATA31D1: Spermatogenesis-associated protein 31D1
SSRIs: selective serotonin reuptake inhibitors
STAT3: Signal transducer and activator of transcription 3
STK32C: Serine/threonine-protein kinase 32C
SVM: Support Vector Machines
TCM: traditional Chinese medicine
TCMSP: Traditional Chinese Medicine Systems Pharmacology Database
TCF4: Transcription factor 4
TGGR: total ginsenoside ginseng root
TIMP4: Metalloproteinase inhibitor 4
TMCO5A: Transmembrane and coiled-coil domain-containing protein 5A
TMEM161B: Transmembrane protein 161B
TMSB4X: Thymosin beta-4
TMT: Tandem Mass Tag
TNF-α: tumor necrosis factor-α
TNRC6B: Trinucleotide repeat-containing gene 6B protein
TRAPPC11: Trafficking protein particle complex subunit 11
TRMT10C: tRNA methyltransferase 10 homolog C
TRPV2: Transient receptor potential cation channel subfamily V member 2
TSLP: Thymic stromal lymphopoietin
Tuba1b: Tubulin alpha-1B chain
TWAS: transcriptome-wide association study
Uba1: Ubiquitin-activating enzyme E1 1
UCH-L1: Ubiquitin carboxyl-terminal hydrolase isozyme L1
UCN2: urocortin 2
UGBBP: Ulm Gene Brain Behavior Project
UFLC/MS-IT-TOF: fast liquid chromatography coupled with ion trap-time of flight mass spectrometry
UFLC/Q-TOF-MS: ultra-fast liquid chromatography/quadrupole-time-of-flight tandem mass spectrometry
UHPLC-MS: ultra high-performance liquid chromatography-mass spectrometry
UHPLC-QTOF-MS: ultra-high performance liquid chromatography quadrupole/time of flight-mass spectrometry
UPLC-MS/MS: ultra performance liquid chromatography tandem mass spectrometry
UPLC-QTOF-MS: ultra-performance liquid chromatography quadrupole/time of flight-mass spectrometry
UPLC-QQQ-MS/MS: ultra-high-performance liquid chromatography-tandem quadrupole mass spectrometry
VAMP-2: Vesicle-associated membrane protein 2
WDR26: WD repeat-containing protein 26
WGBS: Whole-genome bisulfite sequencing
WGS: whole-genome sequencing
WWOX: WW domain-containing oxidoreductase
XAI: Explainable AI
XGBoost: extreme gradient boosting
YOLO: You Only Look Once
ZBTB16: Zinc finger and BTB domain-containing protein 16
Zmynd11: Zinc finger MYND domain-containing protein 11
ZNF184: Zinc finger protein 184
ZNF248: Zinc finger protein 248
ZNF713: Zinc finger protein 713
ZNF804A: Zinc finger protein 804A
2-DE: proteomes by two-dimensional electrophoresis
5-HT: 5-hydroxytryptamine
